# In vitro biological potential of green-synthesized nanoparticles from the Egyptian edible bivalve *Paratapes undulatus* (Born, 1778)

**DOI:** 10.1038/s41598-026-61397-7

**Published:** 2026-08-19

**Authors:** Aida I. El Makawy, Khaled M. Zayed, Dalia M. Mabrouk, Mohamed M. Abd Elrahman, Amal G. Hussien, Cinderella A. Fahmy

**Affiliations:** 1 Cell Biology Department-Biotechnology Research Institute- National Research Centre, 33 El Bohouth St., Dokki, P.O. 12622, Giza, Egypt; 2 Medical Malacology Department, Theodor Bilharz Research Institute, 1 Corniche El-Nile St., Warrak El-Hadar, Imbaba, P.O. 12411,, Giza, Egypt; 3 Biochemistry Dept.- Biotechnology Research Institute, National Research Centre, 33 El Bohouth St., Dokki, P.O. 12622, Giza Egypt; 4 Cancer Biology and Genetics Laboratory, Centre of Excellence for Advanced Sciences, National Research Centre, 33 El Bohouth St., Dokki, P.O. 12622, Giza, Egypt

**Keywords:** Mollusks, Bivalve clams, Metallic nanoparticles, Biological activities, Cytotoxicity, Apoptosis-related genes, DNA fragmentation, Biochemistry, Biological techniques, Biotechnology, Cancer, Chemistry, Drug discovery, Nanoscience and technology

## Abstract

Egypt’s coastal waters host a diverse array of edible bivalves, including clams, mussels, and oysters. Among these, the Mediterranean clam *Paratapes undulatus* (Born, 1778) possesses substantial ecological and nutritional importance. This study investigates the chemical profile and multifunctional biological properties of *P. undulatus* (Pu) ethanolic extract and its green-synthesized nanoparticles (ZnO, Ag, and Se). The biosynthesized NPs were characterized via UV-Vis spectroscopy and transmission electron microscopy (TEM). GC–MS profiling revealed a rich spectrum of bioactive metabolites, including porphyrins, organosilicon compounds, sulfur derivatives, and steroids, which serve as natural stabilizing and capping agents during nanoparticle formation. The in vitro biological activities of the extract and nanoparticles were investigated. Anticancer potential was assessed against human colorectal adenocarcinoma (Caco-2) and hepatocellular carcinoma (HepG-2) cell lines. Results indicated that green synthesis markedly enhanced the extract’s biological performance. Pu–SeNPs exhibited the highest potency, demonstrating superior antioxidant (DPPH, ABTS, NO), antidiabetic, anti-Alzheimer’s, anti-arthritic, and anti-inflammatory activities. Pu–ZnO NPs showed significant bioactivity, though less pronounced than the selenium counterparts. In contrast, Pu–AgNPs displayed minimal improvement over the crude extract. Notably, the apoptosis marker revealed that the unmodified extract was the most effective formulation against Caco-2 cells. However, the biosynthesized nanoparticles exhibited inconsistent efficacy against HepG-2 cells, necessitating further mechanistic investigation. *Paratapes undulatus*-mediated green synthesis, particularly for selenium nanoparticles, represents a promising multifunctional beneficial platform. These findings highlight the potential of marine-derived bivalve extracts in developing sustainable, bio-based nanomedicines to combat oxidative stress, metabolic disorders, neurodegeneration, and selective oncogenic pathways.

**Supplementary Information:**

The online version contains supplementary material available at 10.1038/s41598-026-61397-7.

## Introduction

Marine ecosystems represent a prolific and largely underexploited reservoir of natural products with unique chemical structures^1^. To survive in harsh, highly competitive, and fluctuating marine habitats, marine invertebrates have evolved sophisticated biochemical pathways^2,3^. Among these, mollusks are particularly notable for producing specialized secondary metabolites and defensive enzymes that exhibit significant pharmaceutical potential, including potent antioxidant, antimicrobial, and anti-inflammatory activities^4,5^. However, while the medicinal potential of various marine gastropods (such as *Thais savignyi*, *Trochus erithreus*, and *Cellana rota*) has been increasingly documented in recent literature^5–8^, a vast majority of marine bivalves remain thoroughly unexamined for advanced biomedical applications. This represents a critical missed opportunity; the rapid global emergence of multidrug-resistant pathogens and the high systemic toxicity of conventional, synthetic medications necessitate an urgent, continuous search for novel, biocompatible medicinal agents^9,10^.

Bivalve clams are highly regarded not only as a sustainable food source but also for their rich biochemical profile, including high-quality lean proteins, sulfated polysaccharides, specialized peptides, and unique sterols. *Paratapes undulatus* (Born, 1778), or the undulate venus clam, is a commercially and ecologically significant species inhabiting sandy coastal substrates in tropical and subtropical waters^11^. While baseline investigations such as those conducted by Ghareeb et al.^12^ have successfully characterized the basic antimicrobial and antioxidant profiles of crude *Paratapes undulatus* methanolic extracts, these raw biomolecules face major clinical bottlenecks, including poor structural stability, rapid enzymatic degradation, and inadequate targeted biodelivery^13^. A thorough search of the literature confirms that the biology of the crude extract has been explored; there is an absolute absence of peer-reviewed reports evaluating *Paratapes undulatus* as a biological matrix for metallic or metalloid nanoparticles. The precise chemical reduction kinetics, surface-capping mechanics, and nano-stabilization potential of *P. undulatus* ethanolic metabolites remain entirely unexamined.

Nanoscience offers a revolutionary framework to overcome these limitations by using biological matrices to stabilize and enhance natural products. Biogenic (green) synthesis has emerged as an environmentally benign, reagent-free alternative to traditional chemical synthesis, which frequently relies on toxic reducing agents and leaves hazardous byproducts that compromise clinical biocompatibility^14^. In green nanotechnology, marine biomolecules, which are rich in key functional chemical groups such as hydroxyl (-OH), amine (-NH_2_), and carboxyl (-COOH), play a crucial role in reducing metal ions in the precursor solution to neutral atoms, initiating the nucleation process. Subsequently, the MNPs begin to aggregate, and their stability depends on the bioactive compounds, which act as capping and stabilizing agents to prevent aggregation and improve biocompatibility^15^. To maximize the therapeutic efficacy of *P. undulatus* extracts, this study strategically selects three distinct inorganic platforms: silver (Ag), zinc oxide (ZnO), and selenium (Se). Each possesses unique, complementary biomedical properties: silver nanoparticles are widely studied for their unique physical, chemical, and biological properties^16^. ZnONPs are highly valued for their excellent biocompatibility, low toxicity, sustainability, and cost-effective properties^17^. SeNPs are renowned for their exceptional trace-element safety profile and profound antioxidant and anti-cancer activities, which directly counteract oxidative stress-driven diseases^18^. By synthesizing a triad of Ag, ZnO, and Se nanoparticles using a singular biological matrix, we can directly compare how different inorganic cores interact with and display the clam’s bioactive capping layer, potentially yielding a versatile toolkit of sustainable, eco-friendly therapeutic agents.

while plant-mediated green synthesis remains the most widely used approach, marine-derived bioreductants offer distinct advantages, including: (i) access to unique chemical classes absent in terrestrial plants, such as metallated macrocycles, sulfated polysaccharides, and marine-specific sterols^15^; (ii) the additional layer of bioactivity conferred by the marine-origin capping matrix^16^; and (iii) the potential for sustainable valorization of edible bivalves as by-products of the seafood industry, aligning with circular economy and blue Economy principles^19^. Despite the clear benefits of marine-mediated nanotechnology, a profound knowledge gap persists. While macroalgae and certain microbes have been widely used to synthesize metallic nanoparticles with robust antimicrobial properties^20,21^, a comprehensive search of the literature confirms the absence of peer-reviewed reports evaluating *Paratapes undulatus* or its close taxonomic relatives in nanoparticle formulation. The specific interaction between *P. undulatus* ethanolic metabolites and metallic/metalloid precursors remains entirely unexplored.

To address this gap, the present study was designed to: (1) characterize the chemical profile of the ethanolic extract of P. undulatus soft tissue using GC-MS; (2) utilize this extract as a bioreductant for the green synthesis of Ag, ZnO, and Se nanoparticles; (3) characterize the biosynthesized nanoparticles using UV-Vis spectroscopy and TEM; and (4) comparatively evaluate the in vitro antioxidant, antidiabetic, anti-Alzheimer’s, anti-arthritic, anti-inflammatory, and anticancer activities of the crude extract and its three nanoformulations, thereby establishing a foundational framework for marine-derived nanomedicine.

## Materials and methods

### Biological matrix collection and preparation

Marine clams *Paratapes undulatus* (Born, 1778), Family: *Veneridae*, Genus: *Paratapes*, were collected from Alexandria, Egypt, with the GPS coordinates of 31° 12’ 20.7108’’ N and 29° 55’ 28.2936’’ E., on the Mediterranean coast during the summer of 2024 and spring 2025. The calms were washed thoroughly with seawater and distilled water to remove sand, debris, and surface contaminants. The shell was removed, and approximately 100 g of soft tissue was carefully dissected and stored at − 20 °C until extraction. Before extraction, the soft tissues were thawed and homogenized using a sterile mortar and pestle^8^.

### Preparation of ethanolic extract

The soft tissues of the marine clam were carefully dissected, washed with distilled water, and homogenized. Approximately 100 g of tissue was soaked in 500 mL of 95% ethanol (1: 5) with an initial extract concentration of 0.2 g/mL and kept at room temperature (25 ± 2 °C) in the dark for 72 h with occasional shaking. The mixture was filtered using Whatman No. 1 filter paper with a pore size of 11 μm, and the residue was re-extracted twice to maximize yield. The combined filtrates were concentrated using a rotary evaporator under reduced pressure at 40–45 °C to obtain a semi-solid crude extract, which was then stored in the dark at 4 °C for further analysis^7^.

### Biogenic synthesis of metallic nanoparticles

The green synthesis of silver (AgNPs), zinc oxide (ZnO-NPs), and selenium nanoparticles (SeNPs) was performed using the *P. undulatus* ethanolic extract as a reducing and stabilizing matrix. To achieve experimental reproducibility, all optimization parameters were standardized across three independent batches for nanoparticle type using a constant incubation temperature of 60 °C, continuous magnetic stirring at 500 rpm, an initial reaction pH of 8.5, and a total reaction time of 24 h. The synthesis endpoints were monitored via UV-Vis spectrophotometry until the surface plasmon resonance (SPR) intensity stabilized. For AgNPs preparation, a 10 mL aliquot of the crude extract was mixed with 90 mL of a 1 mM silver nitrate (AgNO) solution. The mixture was stirred in the dark at room temperature until a distinct color transition from pale yellow to dark brown verified reduction. The ZnO-NPs were prepared by introducing a 10 mL volume of the extract dropwise into 90 mL of a 0.01 M zinc acetate solution under continuous agitation. The reaction pH was adjusted to 10.0 using 1 M NaOH to induce a white precipitate, and the slurry was subsequently heated at 60–70 °C for 2 h. For SeNPs, 10 mL of extract was mixed with 90 mL of a 1 mM sodium selenite (Na_2_SeO_3_) solution and incubated under stirring at 40–50 °C until a deep red color, indicating successful selenite reduction. To standardize nanoparticle concentrations for downstream biological profiles, the synthesized nanomaterials were centrifuged at 10,000 rpm for 15 min, repeatedly washed with deionized water and ethanol to eliminate unbound biomolecules, and dried to a constant weight in an oven at 50 °C. The average nanoparticle yield was approximately 1.5 mmol. Purified nanoparticles were resuspended in nanopure water at a stock concentration of 15 mg/mL and stored in airtight glass vials at 2–8 °C^22,23^.

### Physicochemical characterization arrays

The biosynthesized metal nanoparticles (M-NPs) were characterized by UV-Vis spectroscopy using a Shimadzu UV-240 spectrophotometer. The spectra of each biosynthesized M-NP were measured from 200 to 800 nm after the samples were diluted tenfold with deionized water. The biosynthesized M-NPs were analyzed for shape and size using a transmission electron microscope (TEM) model JEM-1230 from Japan. The TEM has a resolution of 0.2 nm, a maximum magnification of 600 × 103, and operates at 200 KV.

### Gas chromatography-mass spectrometry (GC-MS) analysis

To prepare the samples for analysis, 10 mg of the dried *P. undulatus* ethanolic extract and biogenic nanoparticles were individually dissolved in 1 mL of GC-grade ethanol, sonicated for 5 min, and filtered through a 0.22 μm PTFE syringe filter. The chemical constituents were identified using an ultra-trace GC-ISQMS system (Thermo Fisher GmbH) equipped with a TG5sil/MS capillary column (30 m x 0.25 mm x 0.25 mu). A 1 µL diluted sample was introduced via an AI-3000 auto-injector in split mode (1:30) at an injector temperature of 250 °C. The procedural blank was designed to monitor contamination by a mixture of 5mL water and 5mL n-hexane-saturated acetonitrile throughout the entire analytical workflow without matrix. Compound identification was performed by comparing obtained mass spectra and retention indices with entries in the NIST Mass Spectral Library, applying a ≥ 80% match score threshold. All compound assignments were considered tentative, based on spectral matching rather than confirmation with authentic reference standards, in accordance with standard metabolomics reporting practices.

In vitro antioxidant and free radical scavenging characterization

## In vitro antioxidant and free radical scavenging characterization

### Total antioxidant capacity (TAC)

The TAC was measured according to Prieto et al.^24^. A solution comprising 0.6 M sulfuric acid, 28 mM sodium phosphate, and 4 mM ammonium molybdate was combined with 1 mL of extract. After 90 min of incubation at 90 °C, the absorbance at 700 nm was measured against a blank, with ascorbic acid as the reference.

#### Iron-reducing power (IRP)

Oyaizu^25^ methodology, using ascorbic acid as a standard, the iron-reducing power was calculated in µg/mL. Initially, 200 mL of sodium phosphate buffer (pH 6.6) and 1 mL of 1% potassium ferricyanide were combined with 1 mL of the tested extract (1 mg/ml). After 20 min of incubation at 50 °C, 1 mL of 10% trichloroacetic acid was added to the mixture. The mixture was centrifuged at 2000 rpm for 10 min, and 2.5 ml of supernatant and deionized water, plus 1 ml of ferric chloride (0.1%), were mixed. The optical density was measured at 700 nm compared to a blank. Reducing agent of high absorbance.

#### Radical scavenging activity (RSA) via DPPH and ABTS assays

For the DPPH radical assay, 1 mL of the sample was reacted with 2 mL of a 0.1 mM ethanolic DPPH solution^26^. The mixture was incubated in the dark at room temperature for 30 min, and the absorbance was measured at 520 nm.

For the ABTS assay, the inhibition of the 2,2’-azinobis (3-ethylbenzothiazoline-6-sulfonic acid) radical cation was measured according to Arnao et al.^26^. All assays were performed in triplicate, and the radical scavenging activity percentage (RSA%) was calculated. The concentration of 50% RSA (IC50) was determined via non-linear regression analysis. Ascorbic acid was used as a standard for two assays.

### In vitro antidiabetic performance

The in vitro antidiabetic activity was evaluated by measuring inhibition and IC50 values against α-amylase and α-glucosidase, using acarbose as the standard. The α-amylase inhibition was determined according to the specified method. The sample and α-amylase solution (0.5 mg/mL) were incubated at 37 °C for 20 min. Then, a 1% starch solution was added, and incubation continued at the same temperature for 30 min. The reaction was stopped with dinitrosalicylic acid reagent, and absorbance was measured at 540 nm. The α-glucosidase inhibition was assessed by spectrophotometry after incubation with the test samples, read at 405 nm. The analyses were in triplicate, and the percentage of enzyme inhibition was calculated by Inhibition % = ((Ac − As)/Ac) × 100, and IC₅₀ values were obtained via nonlinear regression analysis.

### In vitro anti-Alzheimer’s performance

The percent of acetylcholinesterase (AChE) was measured using Ellman’s technique^27^ using donepezil as the standard drug. The extract (5 µL dissolved in a phosphate buffer (0.1 M, pH 8)) was added to 5 µL of acetylthiocholine (ATCh) (0.5 mM) and 5 µL of 5,5′-dithiobis-2-nitrobenzoic acid (DTNB) (0.03 mM), then mixed and incubated at 30 °C for 10 min. Then, 5 µL of AChE (0.3 U/mL) was added to the initial mixture to start the reaction, and the absorbance was measured at 412 nm. Analyses were conducted in triplicate. Nonlinear regression was used to analyze experimental data.

### In vitro anti-arthritic screening

The anti-arthritic activity was assessed using protein denaturation and proteinase inhibition assays, with diclofenac sodium as the standard^28^. Both assays measured the percentage inhibition of enzyme or protein activity at different concentrations, and IC₅₀ values were calculated. Analyses were performed in triplicate, and the absorption was measured by a spectrophotometer at 660 nm. The formula shown below was used to analyze the inhibitory percentage.

The inhibition % = [Abs control – Abs sample/Abs control] x 100, where Abs stands for absorption.

### In vitro anti-inflammatory screening

The anti-inflammatory potential was assessed by inhibition percentage and IC50 of cyclooxygenase-1 (COX-1), cyclooxygenase-2 (COX-2), and 5-lipoxygenase (5-LOX) using commercial enzyme kits and the manufacturers’ protocols. The percentage of enzyme inhibition in triplicate was calculated using the formula: Inhibition % = (Ac − As/Ac) ×100.

where A < sub> c</sub > is the absorbance of the control and A < sub> s</sub > is the absorbance in the presence of the sample. All assays were conducted in triplicate. Indomethacin and zileuton were used as standard drugs.

### In vitro anticancer characterization

#### Ethical statement and cell culture infrastructure

The study was carried out in accordance with the recommendations of the Committee of Medical Research Ethics of the National Research Center, Egyptian legal requirements, the Declaration of Helsinki, and good laboratory and medical practices (GCP and GLP) Arrive Guidelines.

Various cell lines were used in this study, including Caco-2 and HepG-2, both purchased from the American Type Culture Collections (ATCC, Manassas, VA, USA). Cells were cultured in Roswell Park Memorial Institute Medium-1640 (RPMI-1640), supplemented with 10% fetal bovine serum (FB), 2 mM L-glutamine, 100 U/ml streptomycin sulfate, 100 U/ml penicillin G sodium, and 250 ng/ml amphotericin B. The cells were maintained in humidified 5% CO_2_ at 37 °C. The media was replaced every 2 days, and cells were passaged every 7–8 days.

#### Tumor anti-progression via MTT cytotoxicity assay

The cytotoxicity of Pu ethanolic extract and its nanoparticles against human cancer cells was assessed using the 3-(4, 5-dimethyl-2-thiazolyl)−2,5-diphenyl-2 H-tetrazolium bromide (MTT) assay. Seeding 0.5 × 10^5^ cells per well is typical for a 24-well plate to reach appropriate confluence for treatment after 24 h. Cells were treated with nanoparticles at various concentrations (12.5, 25, 50, and 100 µg/ml in DMSO) per well for 48 h. Control cells received DMSO (maximum concentration 0.1%). After treatment, the media were discarded, and 40 µl of MTT solution per well was added, followed by a 4-hour incubation. The MTT crystals were then dissolved in acidified isopropanol^29^. Photometric readings were taken at 595 nm using a microplate ELISA reader (SunRise, TECAN, Inc., USA). Cell viability was calculated with the following equation: $\:\mathrm{\%}\:\mathrm{Viability}=\left(\frac{\mathrm{Mean}\:O{D}_{\mathrm{t}}-\mathrm{Mean}\:O{D}_{\mathrm{blank}}}{\mathrm{Mean}\:O{D}_{\mathrm{untreated}\:\mathrm{control}}-\mathrm{Mean}\:O{D}_{\mathrm{blank}}}\right)\times\:100.$ The IC50 value was determined as the concentration that results in 50% cell viability.

#### DNA fragmentation analysis via alkaline comet assay

The comet assay was performed under alkaline conditions to assess DNA damage in cultured cells. After treatment with test compounds, cells were harvested, embedded in low-melting-point agarose on pre-coated microscope slides, and lysed in a buffer containing Triton X-100 and DMSO at 4 °C to remove cellular membranes and proteins. The slides were then incubated in an alkaline electrophoresis buffer (pH > 13) to unwind DNA, then electrophoresed at 25 V for 20 min. Following neutralization with Tris buffer, DNA was stained with ethidium bromide or SYBR Green, and comets were visualized using a fluorescence microscope. To ensure statistical validity and reproducibility, a rigorous scoring and image analysis protocol was implemented. For each experimental group, a total of 100 cells were randomly selected and scored across two duplicate slides (50 cells per slide), avoiding gel edges, bubbles, and overlapping structures. Quantitative image analysis was performed using [Open Comet software via ImageJ/CASP software/Comet Assay IV], where individual comets were isolated via automated baseline thresholding. DNA damage was systematically quantified using three primary metrics: tail length (µm), tail DNA percentage (%), and Olive tail moment (OTM). Statistical processing of the comet parameters was performed using [SPSS/GraphPad Prism software]. Data normality was evaluated using the Shapiro-Wilk test. Because comet assay metrics frequently exhibit non-Gaussian distributions, differences between the control and treated groups were analyzed using one-way Analysis of Variance (ANOVA), followed by Duncan’s post hoc test. A p-value ≤ 0.05 was considered statistically significant. All analysis criteria and baseline methodologies were aligned with established protocols described by Ng et al.^30^.

#### Quantitative real-time PCR (RT-qPCR) for apoptotic gene expressions

RNA was extracted from control and treated cells for 48 hours using Triple Xtractor reagent (Grisp, Portugal) according to the manufacturer’s instructions. The quality of RNA was assessed in gel electrophoresis and spectrophotometry. The RNA was then treated with the RNase-free DNase I set (Grisp, Portugal), and cDNA was synthesized using GScript ULTRA (GeneDirex, Inc). Real-time PCR was used to measure the expression levels of apoptotic genes, Bcl-associated X protein (Bax), B cell lymphoma 2 (Bcl-2), and cysteine aspartic acid-specific protease 3 (Caspase-3) using the appropriate primers: Bax F: 5’- CCCGAGAGGTCTTTTTCCGAG − 3’ R: 5’- CCAGCCCATGATGGTTCTGAT-3; Bcl2 F: 5’ ATGTGTGTGGAGAGCGTCAA-3’ R: 5’- GCCGTACAGTTCCACAAAGG − 3; Caspase-3 F: −5’ TGGTTCATCCAGTCGCTTTG − 3’ R: 5’ - CATTCTGTTGCCACCTTTCG-3’; GAPDH F: −5’ AAGGTGAAGGTCGGAG TCAAC-3’ R: 5’ GGGGTCATTGATGGCAACAATA 3’. Primers were obtained from Macrogen (Korea). GAPDH was used as the housekeeping gene. All primers were validated to have amplification efficiencies between the MIQE range of 95–110% (E = 10^(−1/slope) − 1), as determined by standard curve analysis using 5-fold serial dilutions of cDNA. In a 20-µL RT-qPCR reaction volume, 1 µL of cDNA, 1 µL of forward and reverse primers (10 µM each), 10 µL of Xpert Fast SYBR (Uni) 2X master mix (Grisp, Portugal), and nuclease-free water were used. Amplification began with a 2-minute denaturation at 95 °C, followed by 40 cycles of 95 °C for 5 s, 60 °C for 30 s. No-template controls (NTC) and no-reverse-transcriptase controls (NRT) were included in all runs. Following the final amplification cycle, a melting curve analysis was conducted to confirm the amplicon-specificity and the absence of primer dimerization. Relative expression normalization was performed using GAPDH as a housekeeping gene, after verifying that its baseline cycle threshold (Ct) values exhibited no statistically significant variation across the control and all treated experimental groups (*p* > 0.05). The Ct values obtained from the real-time PCR instrument were exported to Microsoft Excel and analyzed using the 2-ΔΔ Ct method as described by Livak and Schmittgen^31^, in strict compliance with the MIQE guidelines for robust and reproducible molecular data reporting.

### Enzymatic apoptosis markers quantification

The effectiveness of biosynthesized M-NPs against apoptotic and anti-apoptotic enzymes (caspase-3 and Bcl-2) in HepG-2 and CACO-2 cancer cells was evaluated using ELISA kits following established procedures^32^.

### Statistical analyses

Data were analyzed using SPSS statistical software for Windows (version 26). Normality and homogeneity of variances were verified using the Shapiro-Wilk and Levene’s tests. Datasets satisfying both assumptions were analyzed via one-way ANOVA, followed by Duncan’s multiple range test for post hoc comparisons. Results are presented as the mean ± standard error of the mean (SEM) from three independent replicates. Statistical significance was defined at *P* ≤ 0.05.

## Results

### Biosynthesized nanoparticle characterization

The yield of *P. undulatus* ethanol extract was approximately 20 g, and the yield of biosynthesized nanoparticles was 41.68% for silver nanoparticles (Ag-NPs), 50.05% for zinc oxide nanoparticles (ZnO-NPs), and 97.09% for selenium nanoparticles (Se-NPs). The biosynthesis and characterization of biosynthesized nanoparticles were confirmed using UV-VIS spectroscopy and Transmission Electron Microscopy (TEM).

### UV-VIS spectroscopic analysis

**Fig. 1:**
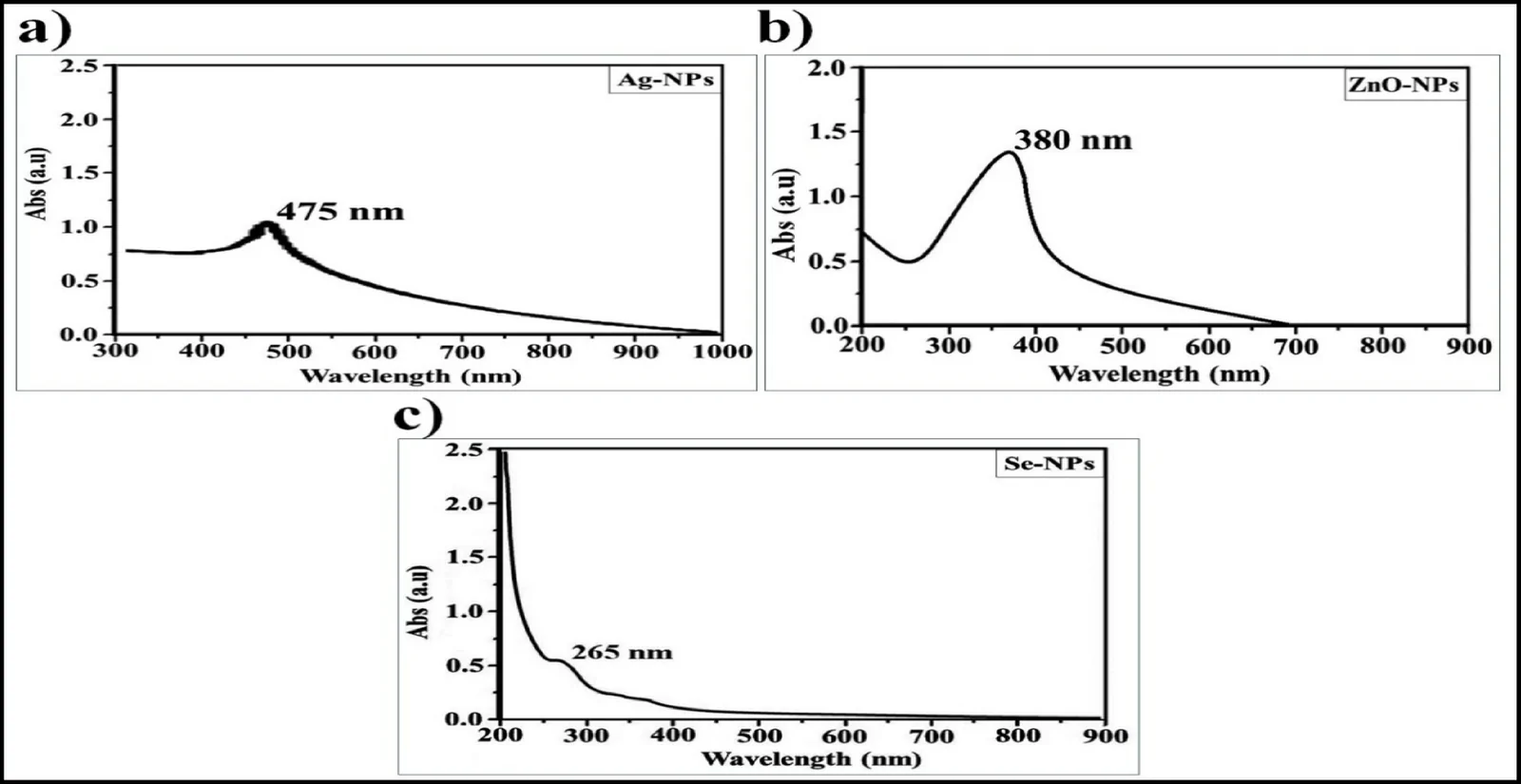
Data of the Ultraviolet-visible (UV-VIS) spectroscopy of the biosynthesized (**a**) Ag-NPs, (**b**) ZnO-NPs, and (**c**) Se-NPs using *Paratapes undulatus* extract.

The formation of the nanoparticles was initially confirmed by ultraviolet-visible (UV-VIS) spectroscopy, which detects the characteristic surface plasmon resonance (SPR) or electronic absorption peaks (Fig. 1). The Ag-NPs exhibited a maximum absorption peak at 475 nm. This peak is characteristic of the SPR phenomenon for silver nanoparticles. The ZnO-NPs displayed a characteristic absorption peak at 380 nm. The Se-NPs showed a primary peak at 265 nm.

### Transmission electron microscopy (TEM) analysis

**Fig. 2:**
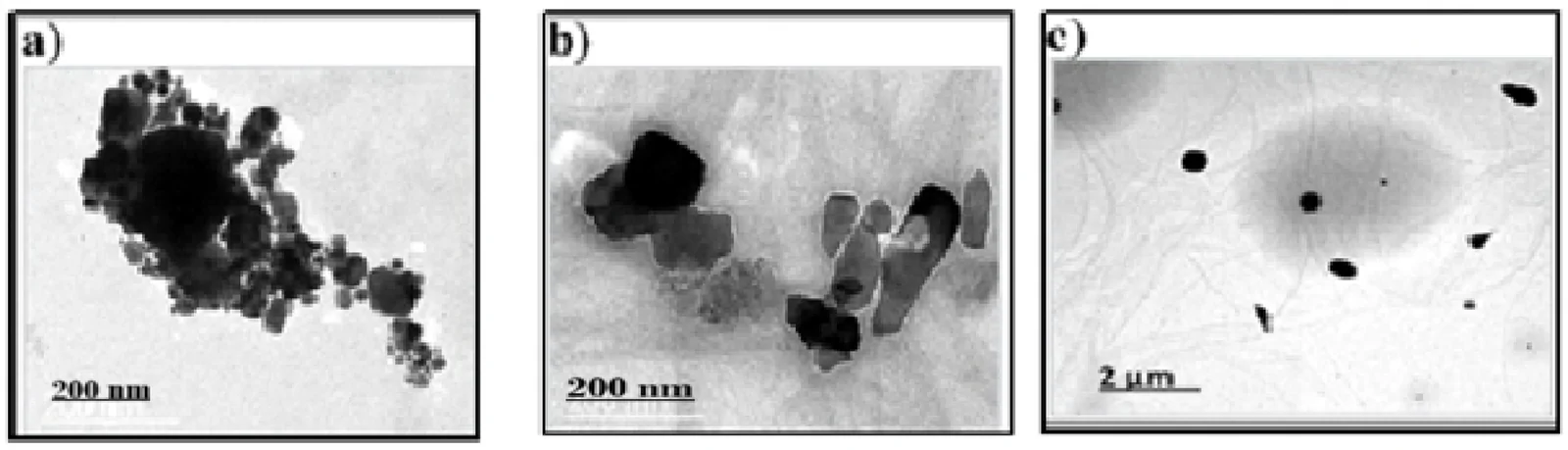
TEM micrographs of the biosynthesized (**a**) Ag-NPs, (**b**) ZnO-NPs, and (**c**) Se-NPs using *Paratapes undulatus* extract.

The TEM analysis of biosynthesized nanoparticles (ZnO-, Ag-, and Se-) provided details on the morphology and relative size (Fig. 2). The micrographs revealed that the AgNPs tend to form aggregated clusters with a size of around 125 nm. The zinc oxide nanoparticles had irregular and non-spherical shapes with a size of around 185 nm. The observed structures appear elongated or rectangular on a scale of 200 nm. The scale bar for this sample is also 200 nm. The selenium nanoparticles appeared as dark, nearly spherical/oval particles with a size of 214 nm. These particles were visible within a lighter, filamentous matrix, suggesting that the NPs may be encapsulated by organic components present in the P. undulatus extract. The scale of the image for the Se-NPs is (2 μm), indicating a larger field of view encompassing the matrix and dispersed nanoparticles.

### GC–MS analysis of *Paratapes undulatus* ethanolic extract

GC–MS analysis was performed to identify the bioactive compounds in the Pu-ethanolic extract and its ZnO-, Ag-, and Se- nanoformulations, as in Fig. 3A–D in the supplementary file. The total ion chromatogram (TIC) of the extract showed a complex profile with over 30 peaks detected within a retention time (RT) range of 5.54 to 52.46 min (Fig. 3A in the supplementary file). Compound identification was achieved by comparing the mass spectra with libraries such as Wiley9 and NIST. The molecular weights and chemical structures of the major constituents are summarized in Table 1 in the supplementary file. The chemical profile was dominated by heterocyclic, organosilicon, porphyrin, sulfur-containing, and steroidal derivatives. Major constituents included 1,5,9,13-tetrathia-3,11-cyclohexadecaediol (10.33%), 7-syn-acetoxy-5-exo-hydroxy-3-exo-methoxycarbonyl-bicyclo [2.2.1] heptane-2,6-carbolactone (8.94%), and 2,4-dibromo deuteroporphyrin IX dimethyl ester (6.16%), which together represented the principal fraction of the extract. Additionally, several metalloporphyrins such as copper tetraphenylporphyrin, (5,10,15,20-tetraphenylporphyrinato) zinc (II), and (2-nitro-5,10,15,20-tetraphenylporphyrinato) nickel (II) were identified, highlighting the presence of metallated macrocycles with strong redox and antioxidant potential. Other notable compounds included organosilicon derivatives (e.g., pentamethyl-pentaphenyl-cyclopentasiloxane), heterocyclic nitrogen compounds (e.g., 1-tert-butyl-1,4-dihydro-5 H-tetrazol-5-one), and sulfur-containing molecules (e.g., 4,8-dimethyl-1,5-dithia-2,6-cyclooctadiene). The coexistence of porphyrins, organosilicons, and sulfur metabolites indicates a synergistic biochemical profile that may contribute to the extract’s observed antioxidant, cytoprotective, and anti-inflammatory activities.

Pu–ZnONPs feature a distinct chemical composition comprising different compounds with retention times 5.02–29.96 min that are summarized in Table 2 in the supplementary file. The chromatogram was dominated by cyclobutylmethanol (43.19%), pentane 3-ethyl-2,4-dimethyl (26.67%), and pregn-5-en-20-one (TMS derivative) (22.90%), collectively representing the major constituents of the nanoparticle formulation. Minor peaks corresponded to 1-bromo-2,2,3-trimethylcyclopropane (1.72%), 3-methyl-3-nitrosobutan-2-one (1.03%), and trace levels of organosilicon (pentaphenyl cyclopentasiloxane, 0.23%), porphyrin zinc complex (5,10,15,20-tetraphenylporphyrinato zinc (II), 0.17%), and cyclohexane carboxamide (0.16%). The presence of both organic and organometallic compounds indicates successful surface capping and stability of ZnO nanoparticles by the extract’s bioactive constituents.

The GC–MS profile of Pu-AgNPs (Fig. 3C in the supplementary file) shows a mixture of low-molecular-weight oxygenated compounds with retention times ranging from 5.10 to 8.35 min that are presented in Table 3 in the supplementary file. The chromatogram was dominated by (S)−4-methyl-γ-butyrolactone (68.41%), indicating its major role as a potential reducing and stabilizing agent during nanoparticle biosynthesis. Other notable constituents included tetraphenylporphyrin to dibromo-titanium (IV) (5.82%), which represents a metalloporphyrin complex with recognized antioxidant and redox-modulating properties, and adipamic acid (2.71%), known for its amide functional groups that may contribute to nanoparticle surface stabilization. Minor compounds such as 7,7-dimethyl-1,4-dioxaspiro [2.4] heptan-5-one, (2R,4R)−2,4-dimethylhexan-1-ol, 1-butanol, and 2-methyl-1,4-butanediol were also detected, reflecting the contribution of small organic molecules.

The chemical profile of Pu–SeNPs (Fig. 3D in the supplementary file) revealed a chemically diverse composition consisting of 30 compounds, with retention times ranging from 5.17 to 8.96 min, as shown in Table 4 in the supplementary file. The spectrum was predominantly characterized by butane, 1-chloro (70.51%), which represents the major peak and may serve as a reducing or solvent-derived component during nanoparticle formation. Other notable compounds included [3,4,2 H₂] cyclopent-3-en-1-ol (6.94%), 6,6-dimethyl-1-ethynyl-7-oxabicyclo [4.1.0] heptan-2-one (4.92%), and tetraphenylporphyrin to dichloro titanium (IV) (5.74%), a metalloporphyrin complex suggesting redox-active coordination that aids nanoparticle stabilization. Additional minor constituents, such as cyclobutylmethanol (2.48%), 2,2-dimethyl-1-nitropropane (0.39%), and 1,1-bis(tri-deuteriomethyl) but-2-yn-1-ol (0.59%), indicate the presence of alcohols, nitro compounds, and bicyclic derivatives contributing to nanoparticle surface modification.

It is important to note that all compound identifications in this study are tentative, based on fragmentation patterns and database matching. Throughout the analysis, the chromatogram is scaled to resolve and identify individual compounds by their characteristic peaks. The software yields comprehensive data for each fragment, including molecular formula, molecular weight, and retention time. Precise detection requires manual optimization of the chromatographic baseline and selective application of peak integration to isolate specific analytes.

### Antioxidant and free radical scavenging activities

The antioxidant and free radical scavenging activities of the *Paratapes undulatus* (Pu) ethanolic extract and its biosynthesized nanoparticles are summarized in Table 1. The crude Pu extract demonstrated moderate antioxidant activity, significantly enhanced following nanoparticle synthesis. Specifically, the Pu-ZnO and Pu-Se nanoparticles exhibited a significant improvement (*p* ≤ 0.05) in Total Antioxidant Capacity (TAC), with respective fold increases of 1.2 and 1.3, and similar enhancements in Iron Reducing Power (IRP). Consistent trends were observed across the free radical scavenging assays. Pu-ZnO and Pu-Se NPs demonstrated significant increases in DPPH inhibition (1.2- and 1.4-fold) and ABTS scavenging (0.8- and 1.3-fold). Furthermore, both formulations significantly boosted nitric oxide (NO) inhibition by 1.3- and 1.4-fold, respectively. In the case of Pu-Ag NPs, all measured parameters showed significant improvements, with an approximately 1-fold increase relative to the crude extract.

**Table 1:** The in vitro antioxidant and free radical scavenging activities of the Ag-NPs, ZnO-NPs, and Se-NPs biosynthesized using *Paratapes undulatus* ethanolic extract compared to ascorbic acid as a standard.

	Antioxidant activity				Free radical scavengers											
	TAC mg	FC	IRP mg	FC	DPPH				ABTS				NO			
					Inhibition (%)	FC	IC50	FC	Inhibition (%)	FC	IC50	FC	Inhibition (%)	FC	IC50	FC
PU-Extract	72.61 ± 0.21e	1.00	54.86 ± 0.21e	1.00	51.27 ± 0.20e	1.00	5.45 ± 0.01c	1.00	55.02 ± 0.20e	1.00	4.78 ± 0.05c	1.00	43.52 ± 0.20e	1.00	6.47 ± 0.03c	1.00
PU-AgNPs	74.06 ± 0.22d	1.02	56.31 ± 0.22d	1.03	52.62 ± 0.20d	1.03	6.71 ± 0.07a	1.02	56.37 ± 0.20d	1.02	5.90 ± 0.06a	1.23	44.87 ± 0.20d	1.03	8.53 ± 0.09a	1.32
PU-ZnONPs	83.50 ± 0.24c	1.15	65.75 ± 0.24c	1.20	61.44 ± 0.23c	1.20	5.75 ± 0.06b	1.18	65.19 ± 0.23c	1.18	5.10 ± 0.05b	1.10	53.69 ± 0.23c	1.23	7.13 ± 0.08b	1.10
PU-SeNPs	**91.85 ± 0.27b**	**1.26**	**74.10 ± 0.27b**	**1.35**	**69.25 ± 0.25b**	**1.35**	**5.10 ± 0.06d**	**0.94**	**73.00 ± 0.25b**	**1.33**	**4.55 ± 0.05d**	**0.95**	**61.50 ± 0.25b**	**1.41**	**6.23 ± 0.07d**	**0.96**
Ascorbic acid	102.37 ± 0.36a	1.41	84.62 ± 0.36a	1.54	79.08 ± 0.33a	1.54	4.47 ± 0.03e	0.82	82.83 ± 0.33a	1.51	4.01 ± 0.05e	0.83	71.33 ± 0.33a	1.64	5.37 ± 0.03e	0.83

Values are presented as the mean ± SE derived from three independent extracts (*n* = 3). Data were analyzed using a one-way ANOVA followed by Duncan’s multiple range test. Different superscript letters within the same column indicate statistically significant differences at *p* ≤ 0.05. *Pu* *Paratapes undulatus*. TAC: total antioxidant capacity, *IRP* iron-reducing power; *FC* Fold change vs. extract. Bolded NPs show the highest activity.

### In vitro antidiabetic activity

The anti-diabetic potential of the *Paratapes undulatus* (Pu) ethanolic extract and its biosynthesized nanoparticles was evaluated against α-amylase, α-glucosidase, and aldose reductase (Table 2). The crude Pu extract showed moderate inhibitory activity, which was significantly enhanced upon nanoparticle formation. Specifically, the Pu–Se NPs exhibited the most substantial improvement (*p* ≤ 0.05), with a 1.44-fold increase in α-amylase inhibition and 1.65-fold increases in both α-glucosidase and aldose reductase inhibition. These results closely approached the efficacy of the standard drugs, Acarbose and Quercetin. Correspondingly, the IC₅₀ values decreased with fold changes of 0.85 and 0.90, respectively, indicating superior potency. Pu–ZnO NPs showed moderate improvements, specifically achieving a 1.19-fold increase in α-amylase inhibition and a 1.31-fold increase in both α-glucosidase and aldose reductase activity compared to the raw extract. IC₅₀ values showing a significant reduction compared to the extract with (0.85 FC) for α-amylase and (0.90 FC) for α-glucosidase. Pu–Ag NPs exhibited the mildest enhancements, with a 1.2-fold increase in α-amylase inhibition and a negligible 1.04-fold change for α-glucosidase and aldose reductase. The IC₅₀ was decreased by (1.04 FC) for α-amylase and (1.14 FC) for α-glucosidase.

**Table 2:** The in vitro antidiabetic activity of Ag, ZnO, and Se nanoparticles biosynthesized using *Paratapes undulatus* ethanolic extract compared to the corresponding standard.

	α-Amylase				α-Glucosidase				Aldose reductase			
	Inhibition %	FC	IC50 (µg/mL)	FC	Inhibition %	FC	IC50 (µg/mL)	FC	Inhibition %	FC	IC50 (µg/mL)	FC
Pu	44.01 ± 0.16e	1.00	4.84 ± 0.02b	1.00	33.27 ± 0.20e	1.00	3.32 ± 0.06c	1.00	41.58 ± 0.25e	1.00	6.47 ± 0.02a	1.00
Pu-Ag NPs	45.10 ± 0.16d	1.20	5.81 ± 0.08a	1.20	34.62 ± 0.20d	1.04	4.73 ± 0.05a	1.42	43.28 ± 0.25d	1.04	5.07 ± 0.02b	0.79
Pu-ZnO NPs	52.16 ± 0.18c	1.19	5.03 ± 0.07b	1.04	43.44 ± 0.23c	1.31	3.77 ± 0.04b	1.14	54.31 ± 0.28c	1.31	4.04 ± 0.01c	0.62
Pu-Se NPs	**63.48 ± 0.22b**	**1.44**	**4.13 ± 0.06c**	**0.85**	**54.73 ± 0.22b**	**1.65**	**3.00 ± 0.03d**	**0.90**	**68.41 ± 0.27b**	**1.65**	**3.21 ± 0.01d**	**0.50**
STD	Acarbose				Quercetin							
	65.87 ± 0.56a	1.49	3.98 ± 0.06c	0.82	61.08 ± 0.33a	1.84	2.68 ± 0.02e	0.81	76.35 ± 0.42a	1.84	2.87 ± 0.02e	0.44

Values are presented as the mean ± SE derived from three independent extracts (*n* = 3). Data were analyzed using a one-way ANOVA followed by Duncan’s multiple range test. Different superscript letters within the same column indicate statistically significant differences at *p* ≤ 0.05. *Pu* *Paratapes undulatus;* *STD* Standard drug; *FC* Fold change vs. extract. Bolded NPs show the highest activity.

### Anti-Alzheimer activity

**Fig. 3:**
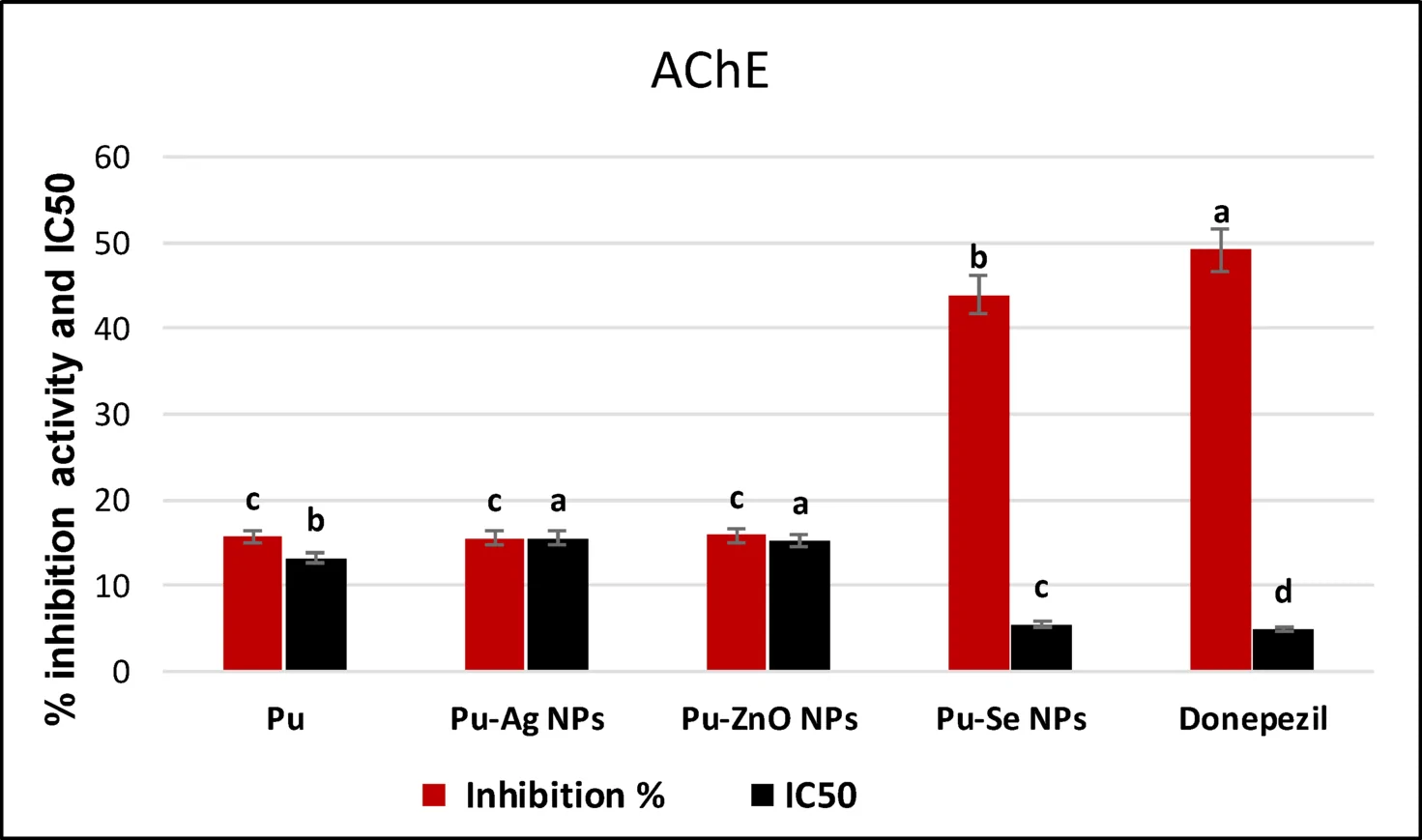
The in vitro anti-Alzheimer’s activity of the Ag, ZnO, and Se nanoparticles biosynthesized using *Paratapes undulatus* ethanolic extract (Pu) is represented by inhibition % and IC50 µg/mL. The values were calculated from a 3-sample and given as mean ± SE. Different letters indicate a significant difference at *p* ≤ 0.05. Donepezil is used as a standard anti-Alzheimer’s drug.

The inhibitory effects of the *Paratapes undulatus* (Pu) extract, and its biosynthesized nanoparticles against Acetylcholinesterase (AChE) are shown in Fig. 3. All nanoparticle formulations demonstrated varying degrees of improvement compared to the raw extract. Pu–Se NPs showed a significant increase in AChE inhibition (˷ 36%) and the most substantial reduction in IC₅₀ (˷ 8 µg/mL). It closely approached the efficacy of the standard drug, Donepezil. Pu–ZnO and Pu–Ag NPs exhibited no significant improvement in AChE inhibitory activity and IC50 compared to the crude Pu extract. Donepezil remained the most potent inhibitor, achieving nearly 50% inhibition with the lowest IC₅₀.

#### Anti-arthritic activity

All nano-formulations enhanced the inhibition of protein denaturation and proteinase (Table 3). Pu–Se NPs showed the greatest enhancement, with a 3.12-fold increase in protein denaturation inhibition and a 3.58-fold increase in proteinase inhibition. Correspondingly, the IC₅₀ for protein denaturation was markedly reduced by 0.34 FC, indicating a substantial increase in potency that nearly matched the standard drug, Diclofenac Sodium. Pu–ZnO NPs showed a 1.02-fold increase in both protein denaturation and proteinase inhibition. However, the IC₅₀ for protein denaturation decreased by 1.19 FC, suggesting slightly lower potency despite the small increase in maximum inhibition. Pu–Ag NPs showed negligible changes in inhibitory activity compared to the raw extract, with fold changes of 0.997 and 0.996 for protein denaturation and proteinase inhibition, respectively. Similar to the ZnO variant, its IC₅₀ for protein denaturation decreased by 1.22-fold.

**Table 3:** The in vitro anti-arthritic activity of the Ag, ZnO, and Se nanoparticles biosynthesized using *Paratapes undulatus* ethanolic extract compared to the corresponding standard.

	Anti-arthritic activity					
	Protein denaturation				Proteinase	
	Inhibition %	FC	IC50 (µg/mL)	FC	Inhibition %	FC
Pu	15.29 ± 0.04^c^	1.00	21.18 ± 0.08^c^	1.00	12.59 ± 0.04^c^	1.00
Pu-Ag NPs	15.25 ± 0.10^c^	0.99	25.89 ± 0.23^a^	1.22	12.55 ± 0.10^c^	0.99
Pu-ZnO NPs	15.57 ± 0.10^c^	1.02	25.26 ± 0.23^b^	1.19	12.87 ± 0.10^c^	1.02
Pu-Se NPs	**47.73 ± 0.17** ^b^	**3.12**	**7.22 ± 0.03** ^d^	**0.34**	**45.03 ± 0.17** ^b^	**3.58**
Diclofenac Sodium	49.73 ± 0.11^a^	3.23	6.91 ± 0.03^d^	0.33	47.03 ± 0.11^a^	3.74

Values are presented as the mean ± SE derived from three independent extracts (*n* = 3). Data were analyzed using a one-way ANOVA followed by Duncan’s multiple range test. Different superscript letters within the same column indicate statistically significant differences at *p* ≤ 0.05. *Pu* *Paratapes undulatus*; *FC* Fold change vs. extract. Bolded NPs show the highest activity.

### Anti-inflammatory activity

The study of anti-inflammatory activity demonstrates that nanoparticle synthesis significantly enhances enzymatic inhibition (Table 4). For COX-1, Pu-Se NPs and Pu-ZnO NPs showed the highest potency with inhibition FCs of 1.88 and 1.87, respectively, while their IC50 FCs dropped to approximately 0.65, indicating higher efficiency at lower concentrations. A similar trend was observed for COX-2, where these two nanoparticles achieved inhibition FCs of roughly 1.80 and IC50 FCs of 0.66. The most improvements occurred in the 5-LOX, where Pu-Se NPs and Pu-ZnO NPs increased the inhibition percent by more than doubling the baseline activity FCs of 2.13 and 2.11. In contrast, Pu-Ag NPs consistently underperformed the baseline across all enzymes, with inhibition FCs ranging from 0.43 to 0.60 and IC50 FCs nearly doubling, suggesting they are less effective than the raw Pu extract.

**Table 4:** The in vitro anti-inflammatory activity of the Ag, ZnO, and Se nanoparticles biosynthesized using *Paratapes undulatus* ethanolic extract compared to the corresponding standard

	Anti-inflammatory activity											
	COX-1				COX-2				5-LOX			
	Inhibition %	FC	IC50	FC	Inhibition %	FC	IC50	FC	Inhibition %	FC	IC50	FC
Pu	22.02 ± 0.08^c^	1.00	15.08 ± 0.14^b^	1.00	24.27 ± 0.08^c^	1.00	10.64 ± 0.01^b^	1.00	17.12 ± 0.08^c^	1.00	22.15 ± 0.07^b^	1.00
Pu-Ag NPs	12.20 ± 0.08^d^	0.55	33.39 ± 0.41^a^	2.21	14.45 ± 0.08^d^	0.60	21.31 ± 0.21^a^	2.00	7.30 ± 0.08^d^	0.43	40.19 ± 0.30^a^	1.81
Pu-ZnO NPs	**41.09 ± 0.14** ^b^	**1.87**	**9.92 ± 0.09** ^c^	**0.66**	**43.34 ± 0.14** ^b^	**1.79**	**7.11 ± 0.05** ^c^	**0.65**	**36.19 ± 0.14** ^b^	**2.11**	**12.24 ± 0.07** ^c^	**0.55**
Pu-Se NPs	**41.50 ± 0.15** ^b^	**1.88**	**9.82 ± 0.09** ^c^	**0.65**	**43.75 ± 0.15** ^b^	**1.80**	**7.04 ± 0.05** ^c^	**0.66**	**36.60 ± 0.15** ^b^	**2.13**	**12.10 ± 0.07** ^c^	**0.02**
	Indomethacin								Zileuton			
STD	57.19 ± 0.13^a^	2.59	7.12 ± 0.03^d^	0.47	59.44 ± 0.13^a^	2.45	5.18 ± 0.02^d^	0.49	52.29 ± 0.13^a^	3.05	8.47 ± 0.03^d^	0.38

Values are presented as the mean ± SE derived from three independent extracts (*n* = 3). Data were analyzed using a one-way ANOVA followed by Duncan’s multiple range test. Different superscript letters within the same column indicate statistically significant differences at *p* ≤ 0.05. *Pu* *Paratapes undulatus*; *STD* Standard drug; *FC* Fold change vs. extract. Bolded NPs show the highest activity.

### Anticancer activity

#### Cytotoxicity of *Paratapes undulatus* extract and its nanoparticles

**Fig. 4:**
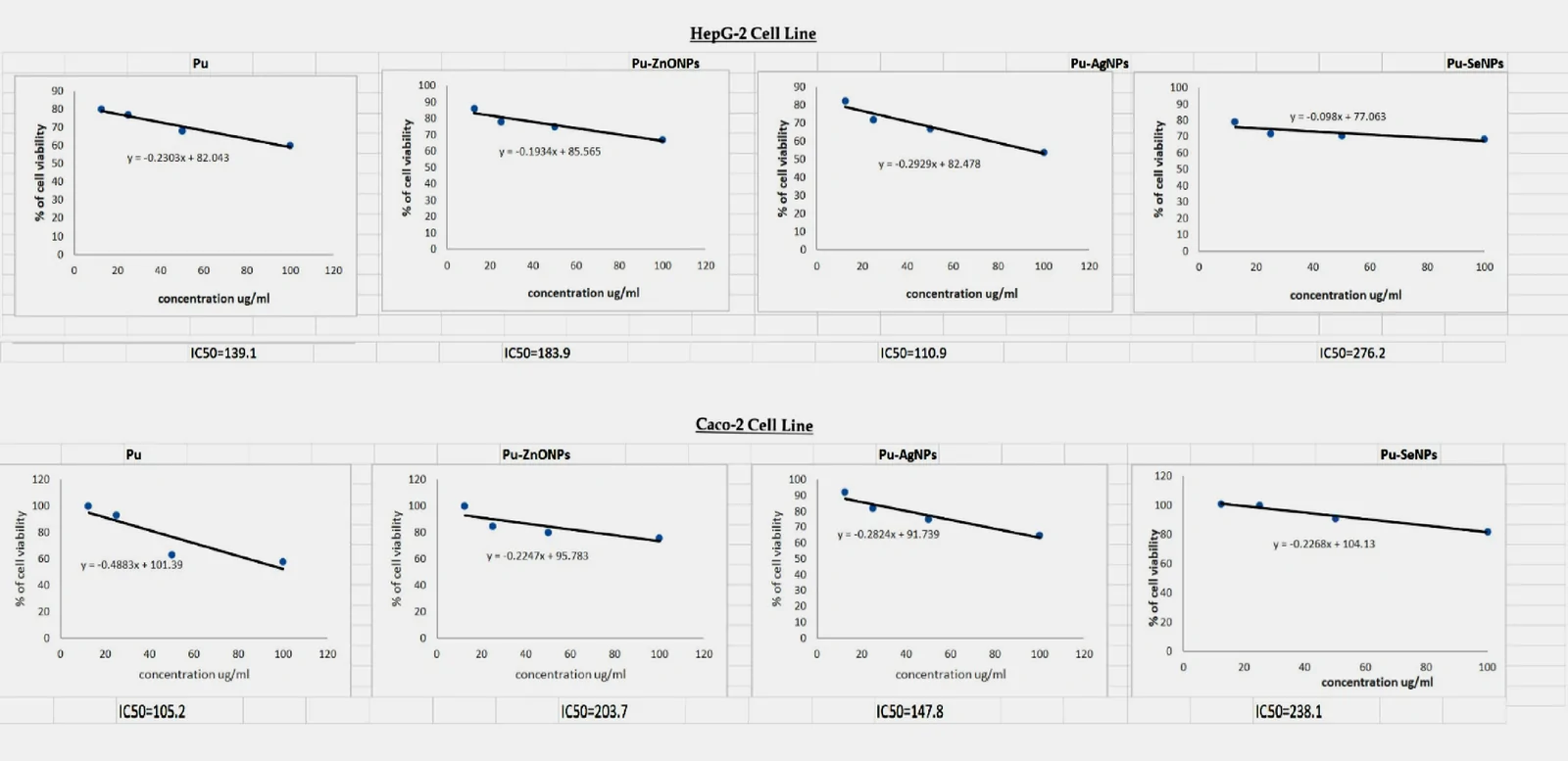
Comparative analysis of the in vitro anticancer potential of Pu extract and its nano-formulations (ZnO, Ag, and Se) across HepG-2 and Caco-2 cells after exposure for 48 h. The figure shows dose-dependent cell viability curves and the calculated IC₅₀ values in HepG2 and Caco-2 cells.

The cytotoxic activity of *Paratapes undulatus* extract, and its biosynthesized nanoparticles against HepG2 and Caco-2 cancer cell lines is illustrated in Fig. 4. Across all treatments, IC₅₀ values exceeded the maximum tested concentration (> 100 µg/mL). In the HepG2 cell line, Pu-AgO NPs exhibited more pronounced inhibitory effects than the raw Pu extract, whereas Pu-ZnO and Pu-Se NPs displayed further reduced cytotoxicity. In contrast, Caco-2 cells revealed a distinct sensitivity profile; the raw Pu extract was the most potent inhibitor, followed by the AgNP formulation. Despite these potency variations, all tested compounds had IC₅₀ values higher than the 100 µg/mL threshold in both cell models.

#### DNA fragmentation

**Fig. 5:**
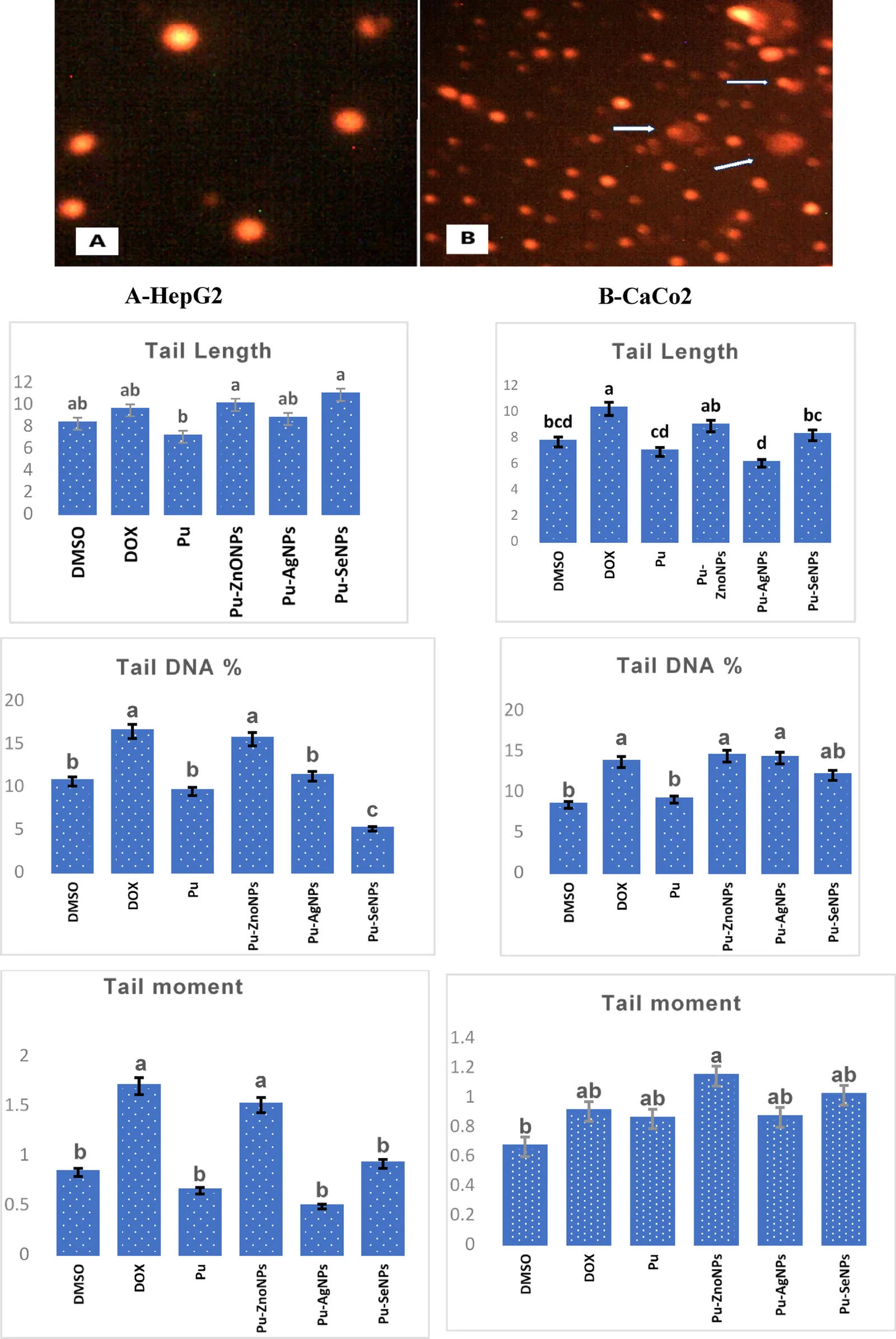
(**A**) Representative images of the comet assay in cell lines showed (a) intact cells and (b) tailed cells (arrow). (**B**) Impact of *Paratapes undulatus* ethanolic extract and its nanoformulations on the DNA in HepG2 and Caco2 cells following a 48-hour exposure.

The DNA-damaging potential of *Paratapes undulatus* extract and its nanoformulations (ZnO, Ag, and Se) was evaluated in HepG2 and Caco-2 cells using the comet assay (Fig. 5). DNA damage was quantified through tail length, tail DNA percentage (tail DNA%), and tail moment (TM), with doxorubicin (DOX) serving as the positive control. In HepG2 cells, ZnO-NPs elicited the most pronounced DNA damage percentage, significantly increasing all comet parameters, approximately as much as DOX. While the Ag-NPs induced moderate DNA fragmentation, Se-NPs exhibited the weakest effect, causing significantly less damage than all other treatments. Conversely, in Caco-2 cells, the genotoxic response varied by parameter. All nanoformulations significantly increased tail DNA% to levels comparable to DOX, suggesting high sensitivity in this cell line. Notably, the raw Pu extract failed to induce significant DNA fragmentation in Caco-2 cells, highlighting a distinct difference in biocompatibility between the extract and its synthesized nanoparticles.

### Analysis of apoptotic gene expression in Caco-2 and HepG2 cells

**Fig. 6:**
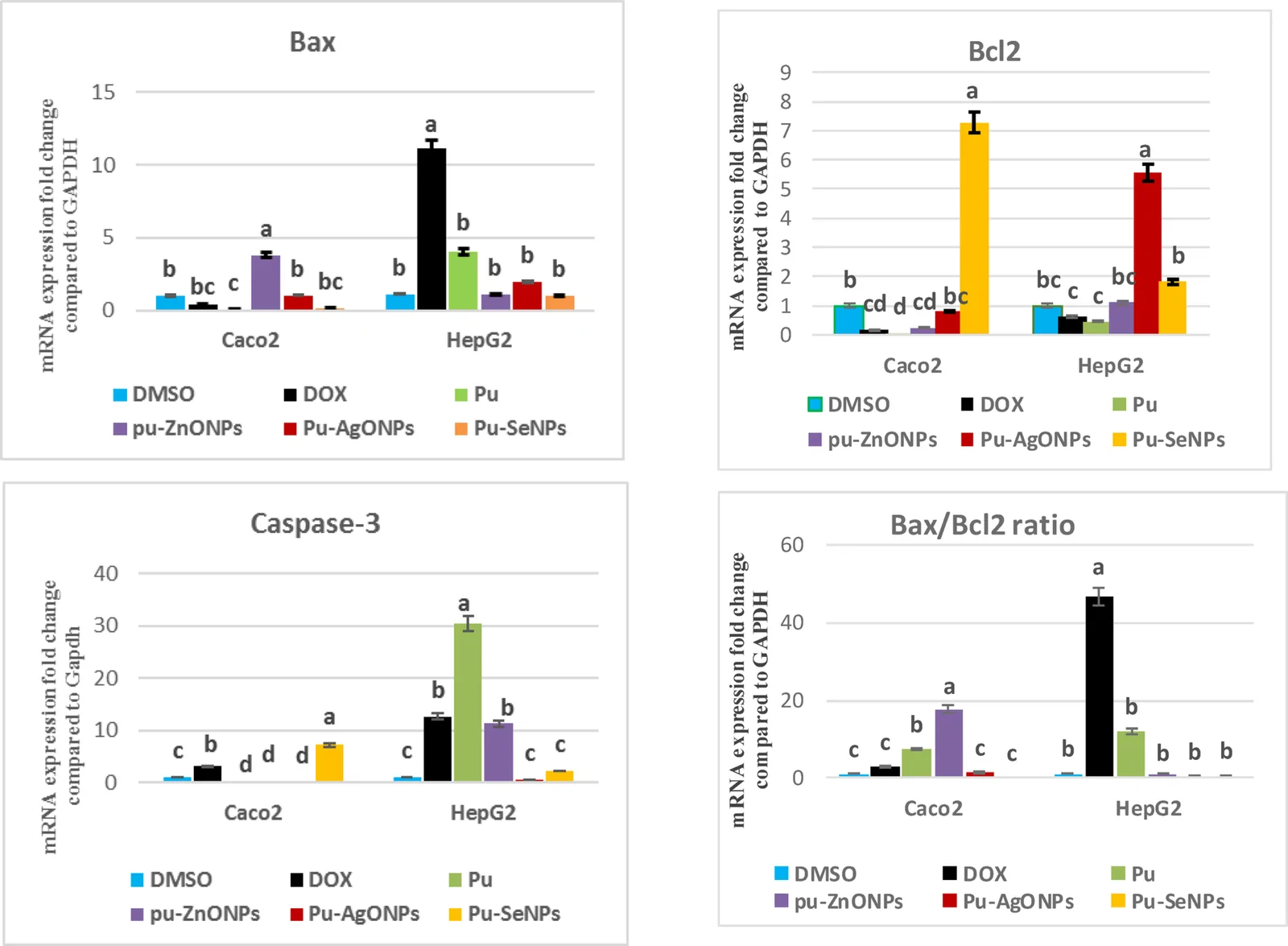
mRNA expression levels of Bax, Bcl2, Caspase-3, and the Bax/Bcl2 ratio in Caco-2 and HepG-2 cells treated with Pu ethanol extract and its nanoformulations (ZnO-, Ag-, and Se-) for 48 h.

The apoptotic potential of the biosynthesized nanoparticles was evaluated by investigating the mRNA expression levels of regulatory genes in Caco2 and HepG2 cell lines (Fig. 6). In Caco2 cells, pu-ZnONPs demonstrated the most potent pro-apoptotic profile, significantly upregulating Bax expression and achieving the highest Bax/Bcl-2 ratio among all tested groups. Conversely, while DOX remained the most effective inducer of the Bax/Bcl-2 ratio in HepG2 cells, the Pu extract alone triggered the most substantial increase in caspase-3 levels within that line. Interestingly, Pu-SeNPs and Pu-AgONPs promoted a marked increase in Bcl2 expression across both cell types, though Pu-SeNPs simultaneously acted as the strongest stimulator of Caspase-3 in Caco-2 cells. These results suggest that while the apoptotic response is treatment-specific, Pu-ZnONPs specifically enhance the intrinsic apoptotic pathway in colorectal cancer cells more effectively than doxorubicin.

### Apoptotic enzymatic analysis

**Fig. 7:**
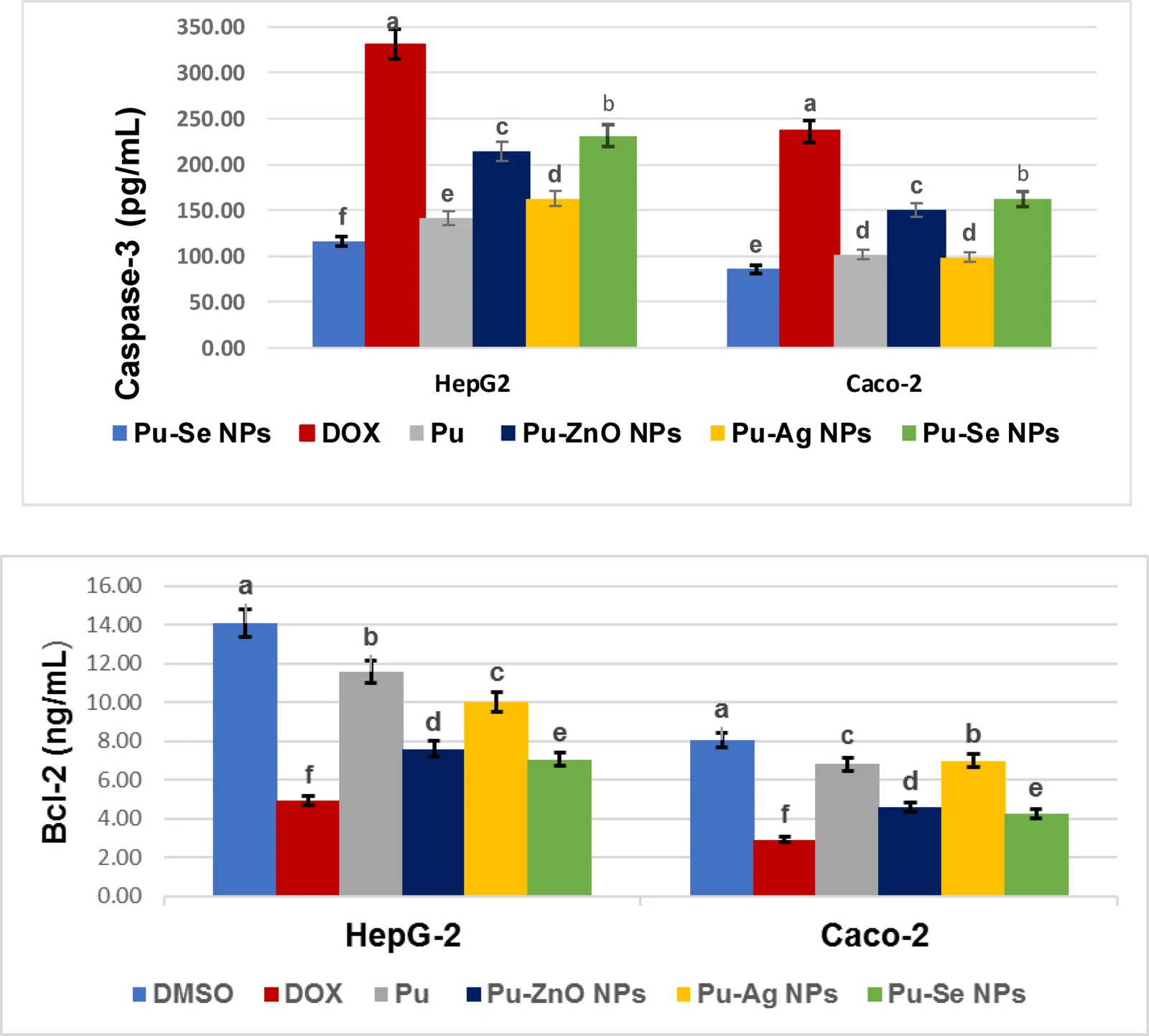
The enzymatic apoptotic markers Caspase-3 and Bcl2levels in HepG-2 and Caco-2 cells treated with Pu ethanol extract and its nanoformulations (ZnO-, Ag-, and Se-) for 48 h.

The effects of *Paratapes undulatus* ethanolic extract and its biosynthesized nanoparticles on apoptotic enzymatic markers (Caspase-3 and Bcl-2) were evaluated in HepG-2 and Caco-2 cell lines, as seen in Fig. 7. The results revealed clear apoptosis in all treated groups compared with the control. In both cell types, treatment significantly (*P* ≤ 0.05*)* increased Caspase-3 activity while concomitantly suppressing the anti-apoptotic protein Bcl-2. The Pu-Se-NPs demonstrated the highest pro-apoptotic potency, yielding Caspase-3 levels of 231.35 pg/mL in HepG-2 and 162.11 pg/mL in Caco-2 cells, while effectively reducing Bcl-2 expression to 7.07 ng/mL and 4.27 ng/mL, respectively. While doxorubicin maintained the highest levels of caspase-3 activation and the lowest Bcl-2 expression overall, the biosynthesized nanoparticles, particularly the selenium, displayed significant potential in the enzymatic machinery of programmed cell death.

## Discussion

The marine bivalve *Paratapes undulatus* represents a valuable source of biologically active compounds, characterized by a diverse chemical profile that includes porphyrin derivatives, steroid-like metabolites, sulfur-containing compounds, and other bioactive constituents. These compounds have been associated with antioxidant, anti-inflammatory, neuroprotective, and anticancer activities to modulate oxidative stress and inflammatory pathways^33–35^. Beyond their intrinsic biological functions, these phytochemicals play a critical role in the green synthesis of silver (Ag), zinc oxide (ZnO), and selenium (Se) nanoparticles by acting as natural reducing, capping, and stabilizing agents. The resulting biogenic nanoconjugates exhibit enhanced physicochemical stability and biological activity, providing a promising platform for translating marine-derived bioactives into multifunctional nanomedicines^36,37^.

The biological performance of nanoparticles is strongly influenced by their physicochemical characteristics, particularly particle size, morphology, aggregation state, and surface chemistry. In the present study, the synthesized nanoparticles exhibited particle sizes ranging from approximately 125 to 214 nm. At this size range, cellular uptake is expected to occur predominantly through endocytic pathways such as macropinocytosis and phagocytosis, facilitating intracellular delivery of bioactive ions and surface-associated biomolecules. Despite their relatively large hydrodynamic size and aggregated morphology, the nanoparticles maintained substantial biological activity, likely due to the presence of the *P. undulatus*-derived bio-organic corona and their high effective surface area^38^. Notably, the filamentous organic matrix observed surrounding the SeNPs appears to provide both steric stabilization and electrostatic repulsion, minimizing irreversible aggregation and promoting colloidal stability. This natural capping layer may also act as a structural template during nanoparticle formation, contributing to controlled particle growth and improved reproducibility.

The biogenic synthesis of nanoparticles (NPs) mediated by *Paratapes undulatus* extract represents a promising approach for enhancing the biomedical applicability of marine-derived bioactive compounds through nanoparticle formulation. Rather than serving as passive carriers, the distinct metallic cores (Se, ZnO, and Ag) interact dynamically with the *P. undulatus* organic matrix, yielding distinct physicochemical profiles. These configurations dictate their unique thermodynamic, enzymatic, and cellular activities across antioxidant, antidiabetic, neuroprotective, anti-inflammatory, and selective anticancer pathways. The pronounced superiority of Pu-Se NPs over Pu-ZnO and Pu-Ag NPs underlines a crucial biochemical synergy between the elemental selenium core and the biogenic capping layer. Conventional chemical synthesis often suffers from rapid, unconstrained particle aggregation driven by high surface energy, which drastically reduces active surface area. In contrast, the *P. undulatus* extract provides a sophisticated, naturally occurring filamentous organic matrix that acts as an optimal steric and electrostatic barrier, maintaining long-term colloidal stability^39^. This structural preservation directly amplifies selenium’s intrinsic redox-scavenging capabilities. The organic capping ligands facilitate efficient electron or hydrogen atom transfer to neutralize reactive oxygen species (ROS) more effectively than the more rigid coordination spheres of ZnO or Ag nanoparticles^40–42^. This observation aligns with El-Saber et al.^43^, demonstrating that the architectural arrangement of the biogenic capping layer, rather than core elemental concentration alone, governs radical-scavenging kinetics.

When transitioning from free-radical scavenging to complex, multi-factorial pathologies, the nanoformulations demonstrated a clear capacity for targeted enzyme modulation. The significant enhancement of *P. undulatus* extract’s antidiabetic potential via Pu-Se and Pu-ZnO functionalization points toward a multi-target therapeutic mechanism. While raw marine extracts often moderate non-specific enzyme inhibition, nanoformulation markedly optimizes the inhibition kinetics against key carbohydrate-hydrolyzing enzymes α-amylase, α-glucosidase, and aldose reductase) alongside the sorbitol pathway driver, aldose reductase. This potency is attributed to a dual-action biochemical pathway. In previous studies, Selenium ions have been shown to act as insulin mimetics, activating downstream glucose transport. Zinc ions serve as critical structural cofactors that support pancreatic beta-cell architecture and insulin secretory mechanisms^44–47^. Consequently, the nanoformulation acts as a molecular shield, mitigating glucose-induced oxidative stress and protecting pancreatic tissue from chronic glucotoxicity^48^.

A parallel synergistic trend was observed in anti-Alzheimer assays; Pu-Se and Pu-ZnO NPs inhibited acetylcholinesterase (AChE) at levels approaching the clinical standard, donepezil. This exceptional potency represents a drastic improvement over traditional, crude marine extracts. The underlying neuroprotective mechanism relies on modulating cholinergic balance by suppressing AChE expression, while upregulating endogenous antioxidant defense systems to alleviate microglial oxidative stress^49^. This implies that these biogenic nanoparticles can exert synergistic effects against cholinesterase activity, a potential target in neurodegenerative diseases^50^.

The anti-arthritic activities of the *Paratapes undulatus*-based formulations were strongly influenced by nanoparticle composition, with Pu-Se NPs exhibiting superior efficacy compared to Pu-ZnO and Pu-Ag NPs. This enhanced activity is likely attributable to selenium’s ability to simultaneously modulate oxidative stress and inflammatory signaling pathways by activating antioxidant selenoenzymes and suppression of NF-κB-mediated responses, resulting in effective inhibition of iNOS, COX-2, and 5-LOX^51–53^. These findings are consistent with previous reports demonstrating the potent anti-inflammatory properties of selenium nanoparticles and suggest a broader multi-target mechanism of action. Pu-ZnO NPs showed moderate activity, in agreement with studies reporting ZnO nanoparticle-mediated inhibition of protein denaturation and inflammatory mediators^54^, which comparatively lower efficacy of Pu-Ag NPs contrasts with published report of anti-arthritic AgNPs^55^, highlighting the importance of nanoparticle source, surface chemistry, and capping biomolecules in determining biological performance.

The anticancer activity of *Paratapes undulatus*-derived nanoparticles appears to be governed by their ability to modulate apoptotic signaling and cellular stress responses. Although Pu-AgNPs exhibited the strongest cytotoxicity toward HepG-2 cells and Pu-ZnONPs were most effective against Caco-2 cells, these differences likely reflect cell type-specific susceptibilities to nanoparticle-induced oxidative stress and mitochondrial dysfunction rather than a uniform mechanism of action. The increased Bax/Bcl-2 ratio and activation of Caspase-3 observed in both cell lines indicate that all nanoformulations primarily trigger the intrinsic mitochondrial apoptotic pathway, consistent with previous reports on biogenic metal nanoparticles^56,57^. However, the moderate cytotoxicity of Pu-SeNPs despite pronounced apoptotic-enzyme activation suggests that induction of pro-apoptotic signaling does not necessarily translate into immediate cell death. Similar discrepancies have been reported in cancer cells capable of mounting adaptive survival responses, including transient upregulation of anti-apoptotic proteins and antioxidant defenses that delay apoptosis execution^58^. This may explain the observed disconnect between DNA damage, apoptotic gene activation, and short-term cell viability. The superior activity of Pu-ZnONPs against Caco-2 cells is in agreement with studies demonstrating enhanced ROS generation and mitochondrial impairment by ZnO nanoparticles in colorectal cancer models, whereas the strong effect of Pu-AgNPs against HepG-2 cells supports previous evidence of AgNP-mediated apoptosis in hepatocellular carcinoma^59^. Collectively, these findings suggest that the selective cytotoxicity of the nanoformulations arises from differences in how individual cancer cell types process oxidative stress, DNA damage, and apoptotic signaling. Although normal cell lines were not evaluated in the present study, previous studies on marine-derived biogenic nanoparticles have reported preferential toxicity toward malignant cells while sparing non-transformed cells, an effect often attributed to natural surface-capping biomolecules that improve biocompatibility and cellular selectivity^60^.

In summary, the pronounced pharmacological activities of the synthesized nanoformulations are driven by a synergistic nano-bio interface that correlates their unique physicochemical properties with specific biological targets. The organic capping layer, rich in electron-donating porphyrins, sulfur metabolites, and lactones acts as a direct free-radical scavenger and anti-inflammatory shield. Concurrently, the core materials have distinct, complementary modalities: Pu-SeNPs leverage essential metalloid biochemistry to upregulate endogenous antioxidant enzymes; Pu-ZnONPs exploit semiconductor wide-bandgap properties to induce cytotoxic, reactive oxygen species (ROS)-mediated pathways; and Pu-AgNPs exert potent structural and enzymatic disruption via controlled silver Ag+ release. Together, this tripartite mechanism combining size enhancement, core-specific reactivity, and biomolecular capping underpins the enhanced therapeutic efficacy observed across the nanoformulations. This study identifies Egyptian bivalve *Paratapes undulatus* as a novel, scalable bio-factory for the green synthesis of multifunctional nanoparticles, bridging the gap between food-industry valorization and nanomedicine. By transitioning a locally significant edible species into a high-value medical precursor, this research aligns with ‘Blue Economy’ principles of sustainability.

## Limitations

Although the present study provides preliminary insights, several limitations should be acknowledged. First, the biological evaluation was restricted to in vitro models (Caco-2 and HepG2), which, while providing essential foundational data, do not fully replicate the complexity of in vivo systems, particularly with respect to biodistribution, metabolic clearance, immune responses, and physiological barriers. Consequently, the observed cytotoxic and bioactivity profiles should be interpreted as preliminary leads rather than confirmed therapeutic outcomes.

Second, the physicochemical characterization of the synthesized nanoparticles remains incomplete. Although nanoparticle formation was confirmed using UV–Vis spectroscopy and TEM analysis, the absence of complementary techniques such as DLS and zeta potential measurements limits a comprehensive assessment of hydrodynamic size distribution, surface charge, and colloidal stability. In addition, FTIR and XRD analyses were not performed, preventing definitive characterization of surface functional groups, capping mechanisms, and crystalline structure. Therefore, these properties were inferred based on available data and previously reported marine-mediated nanoparticle systems.

Third, mechanistic interpretations were largely inferential. Although the results suggest involvement of mitochondrial-mediated apoptotic pathways and oxidative stress mechanisms, these were not directly validated using specific assays. In particular, ROS production, mitochondrial membrane potential disruption, nanoparticle internalization pathways, intracellular trafficking, and release kinetics from the capping matrix were not experimentally investigated. As such, the mechanistic basis of the observed biological effects remains putative.

While the inhibition of acetylcholinesterase suggests potential relevance to neurodegenerative applications, the ability of the synthesized nanoparticles to cross the blood–brain barrier (BBB), as well as their in vivo neurotoxicity and long-term neurological safety, remain uninvestigated. Comprehensive pharmacokinetic and neurodistribution studies are therefore required to evaluate their CNS targeting potential and safety profile.

Fourth, formulation stability and reproducibility were not assessed. No data were generated regarding long-term storage stability, aggregation behavior, sedimentation, protein corona formation, or colloidal stability in biologically relevant media. Additionally, variability associated with marine-derived extracts, including seasonal and geographical fluctuations as well as potential heavy metal contamination in bivalves, may influence nanoparticle synthesis, morphology, and capping efficiency, thereby limiting scalability and standardization for translational applications.

Finally, safety, pharmacokinetic, and environmental considerations remain unaddressed. While Ag and ZnO nanoparticles may release Ag⁺ and Zn²⁺ ions, contributing to cytotoxic effects independent of nanoparticle-specific mechanisms, ion release was not quantified. Moreover, the ability of the synthesized nanoparticles to cross the blood–brain barrier, their in vivo toxicity, pharmacokinetics, and long-term neurotoxicity remain unknown. From an environmental perspective, the potential accumulation of marine-based nanomaterials in aquatic ecosystems and filter-feeding organisms raises concerns regarding ecotoxicological impact, necessitating further comprehensive assessment prior to large-scale or environmental applications.

## Conclusion

This preliminary investigation highlights the marine bivalve *Paratapes undulatus* as a promising bioresource for the green synthesis of multifunctional metal-based nanoparticles. Our results demonstrate that this eco-friendly synthesis approach can yield platforms exhibiting antioxidant, antidiabetic, antiarthritic, anti-inflammatory, and antiproliferative activities in various in vitro models. These findings suggest that *P. undulatus* and its biosynthesized nanoformulations may serve as a foundation for future research into therapeutic agents. However, it is essential to emphasize that these results are preliminary and confined to in vitro assessments. Extensive in vivo studies, comprehensive safety evaluations, and detailed mechanistic investigations are necessary before any conclusions can be drawn regarding their safety profiles or clinical translational potential.

## Supplementary Information

Below is the link to the electronic supplementary material.

Supplementary Material 1

## Data Availability

All data supporting the findings of this study are available within the paper.

## References

[1] Ferreira et al. DHA-Derived Specialized Pro-Resolving Mediators, Biosynthetic Pathways, Synthetic Approaches, and Their Role in Inflammation. *Molecules* **27** (2022). 10.3390/molecules27051677 PMC8912121 35268778

[2] Sarwat, M. S. Nutritional benefits of marine molluscs: A comprehensive review. *Int. J. Zool. Investig.* **9**, 408–414. 10.33745/ijzi.2023.v09i02.045 (2023).

[3] Habib, M. R. et al. Thais savignyi tissue extract: bioactivity, chemical composition, and molecular docking. *Pharm. Biol.* **60**, 1899–1914. 10.1080/13880209.2022.2123940 (2022). 36200747 10.1080/13880209.2022.2123940 PMC9553184

[4] Al, N. Antioxidants obtained from marine sources, Mar. Antioxidants. *Acad. Press.* 45–56. (2023).

[5] Moruf, R. O., Ogunbambo, M. M., Taiwo, M. A. & Afolayan, O. A. Marine bivalves as a dietary source of high-quality lipid: a review with special reference to natural long chain polyunsaturated fatty acids. *Food Sci. Technol.*10.15835/buasvmcn-fst:2020.0064 (2021).

[6] Zayed, K. M. et al. Biological activity, chemical profiling and molecular docking of tissue extracts of the sea snail Trochus erithreus. *J. Appl. Pharm. Sci.* **13**, 199–210. 10.7324/JAPS.2023.119765 (2023).

[7] Habib, M. R. et al. In vitro anticancer and antimicrobial activities of copper-zinc superoxide dismutase purified from Cellana rota snail. *Egypt. J. Chem.* **66**, 1–10. 10.21608/EJCHEM.2023.200467.7736 (2023).

[8] Abdelhady, A. A., Ahmed, M. S., Samy-Kamal, M. & Hussain, A. M. Assessment of ocean acidification impact on gastropod shells using geometric morphometrics. *Water Air Soil Pollut.* **235**, 810. 10.1007/s11270-024-07623-2 (2024).

[9] Wynendaele, E. et al. Sustainability in drug discovery, Med. *Drug Discov*. **12**, 100107. 10.1016/j.medidd.2021.100107 (2021).

[10] Eghianruwa et al. Bioactive Peptides from Marine Molluscs—A Review. *Int. J. Biochem. Res. Rev.* **27**, 1–12 (2019).

[11] Shukhairi, S. S. et al. Microplastics Isolated from Saltwater Clam Paratapes undulatus from Wet Market at Shah Alam, Selangor, Malaysia. *Malaysian J. Fundam Appl. Sci.* **20**, 89–99. 10.11113/mjfas.v20n1.2893 (2024).

[12] Ghareeb, M. A., Hamdi, S. A. H., Fol, M. F. & Ibrahim, A. M. Chemical characterization, antibacterial, antibiofilm, and antioxidant activities of the methanolic extract of *Paratapes undulatus* clams (Born, 1778). *J. Appl. Pharmacol. Sci.* **12** (05), 219–228 (2022).

[13] Lv, Y. et al. Nano-Drug Delivery Systems Based on Natural Products. *Int. J. Nanomed.* **19**, 541–569. 10.2147/IJN.S443692 (2024). 10.2147/IJN.S443692 PMC10802180 38260243

[14] Roychoudhury, A. Yeast-mediated Green Synthesis of Nanoparticles for Biological Applications. *Indian J. Pharm. Biol. Res.* **8**, 26–31. 10.30750/ijpbr.8.3.4 (2020).

[15] Ali, M. A. et al. Advancements in plant and microbe-based synthesis of metallic nanoparticles and their antimicrobial activity against plant pathogens. *Nanomaterials* **10**, 1–24. 10.3390/nano10061146 (2020). 10.3390/nano10061146 PMC7353409 32545239

[16] Rizki, I. N. & Klaypradit, W. Utilization of marine organisms for the green synthesis of silver and gold nanoparticles and their applications: A review. *Sustainable Chem. Pharm.* **31**, 100888. 10.1016/j.scp.2022.100888 (2023).

[17] Asif, N., Amir, M. & Fatma, T. Recent advances in the synthesis, characterization and biomedical applications of zinc oxide nanoparticles. *Bioprocess Biosyst. Eng.* **46** (10), 1377–1398. 10.1007/s00449-023-02886-1 (2023). 37294320 10.1007/s00449-023-02886-1 PMC10251335

[18] Xia, I. F. et al. Selenium nanoparticles (SeNPs) immunomodulation is more than redox improvement: Serum proteomics and transcriptomic analyses. *Antioxidants (Basel)* **11**(5), 964. 10.3390/antiox11050964 (2022). 35624828 10.3390/antiox11050964 PMC9137598

[19] Negm, M. A. et al. Green synthesis of silver nanoparticles using marine algae extract and their antibacterial activity. *Middle East. J. Appl. Sci.* **8** (3), 957–970 (2018).

[20] Jafar, Z. N. & Athbi, A. M. Biosynthesis of silver nanoparticles using ethanolic extract of the marine alga Enteromorpha intestinalis and evaluation of their antibacterial activity. *Egypt. J. Aquat. Biology Fisheries*. **28** (2), 735–756 (2024).

[21] Aboulthana, W. M. et al. Evaluation of antioxidant efficiency of *Croton tiglium* L. Seeds extracts after incorporating silver nanoparticles. *Egypt. J. Chem.* **62**, 181–200. 10.21608/EJCHEM.2018.4960.1442 (2019).

[22] Salayova, A. et al. Green synthesis of silver nanoparticles with antibacterial activity using various medicinal plant extracts: morphology and antibacterial efficacy. *Nanomaterials (Basel)*. **11** (4), 1005. 10.3390/nano11041005 (2021). PMID: 33919801; PMCID: PMC8070782. 33919801 10.3390/nano11041005 PMC8070782

[23] El-Naggar, H. M., Shehata, A. M. & Morsi, M.-A. Micropropagation and GC–MS analysis of bioactive compounds in bulbs and callus of white squill. *In Vitro Cell. Dev. Biol. Plant* **59**, 154–166. 10.1007/s11627-023-10333-9 (2023).

[24] Prieto, P., Pineda, M. & Aguilar, M. Spectrophotometric quantitation of antioxidant capacity through the formation of a phosphomolybdenum complex: specific application to the determination of vitamin e. *Anal. Biochem.* **269**, 337–341. 10.1006/abio.1999.4019 (1999). 10222007 10.1006/abio.1999.4019

[25] Oyaizu, M. Studies on products of browning reaction. Antioxidative activities of products of browning reaction prepared from glucosamine. *Japanese J. Nutr. Diet.* **44**, 307–315. 10.5264/eiyogakuzashi.44.307 (1986).

[26] Arnao, M. B., Cano, A. & Acosta, M. The hydrophilic and lipophilic contribution to total antioxidant activity. *Food Chem.* **73**, 239–244. 10.1016/S0308-8146(00)00324-1 (2001).

[27] Paun, G., Neagu, E., Moroeanu, V., Albu, C. & Savin, S. Chemical and bioactivity evaluation of eryngium planum and cnicus benedictus polyphenolic-rich extracts. *Biomed. Res. Int.*10.1155/2019/3692605 (2019). 30993111 10.1155/2019/3692605 PMC6434295

[28] Alamgeer, A. M., Uttra, U. H. & Hasan Anti-arthritic activity of aqueous-methanolic extract and various fractions of Berberis orthobotrys Bien ex Aitch, BMC Complement. *Altern. Med.* **17**, 1–16. 10.1186/s12906-017-1879-9 (2017). 10.1186/s12906-017-1879-9 PMC5516381 28720131

[29] Hansen, M. B., Nielsen, S. E. & Berg, K. Re-examination and further development of a precise and rapid dye method for measuring cell growth/cell kill. *J. Immunol. Methods* **119**, 203–210. 10.1016/0022-1759(89)90397-9 (1989). 2470825 10.1016/0022-1759(89)90397-9

[30] Ng, C. T. et al. Zinc oxide nanoparticles exhibit cytotoxicity and genotoxicity through oxidative stress responses in human lung fibroblasts and Drosophila melanogaster. *Int. J. Nanomed.* **12**, 1621–1637. 10.2147/IJN.S124403 (2017). 10.2147/IJN.S124403 PMC5339013 28280330

[31] Livak, K. J. & Schmittgen, T. D. Analysis of relative gene expression data using real-time quantitative PCR and the 2(-Delta Delta C(T)) Method. *Methods* **25**, 402–408. 10.1006/meth.2001.1262 (2001). 11846609 10.1006/meth.2001.1262

[32] Pandey, P., Khan, F., Alzahrani, F. A., Qari, H. A. & Oves, M. A novel approach to unraveling the apoptotic potential of rutin (bioflavonoid) via targeting jab1 in cervical cancer cells. *Molecules*10.3390/molecules26185529 (2021). 34577000 10.3390/molecules26185529 PMC8472561

[33] Boulajfene, W. Nutritional and health benefits of marine mollusks. In *Marine Biochemistry* 435–453 (Taylor & Francis, 2022).

[34] Ahmed, A., Omar, W., El-Asmy, H., Abouzeid, L. & Fadda, A. Docking studies, antitumor and antioxidant evaluation of newly synthesized porphyrin and metalloporphyrin derivatives. *Dye Pigment*. **183**, 108728. 10.1016/j.dyepig.2020.108728 (2020).

[35] Wang, T., Shi, W., Mao, Z., Xie, W. & Wan, G. Neuroprotective mechanisms of red algae-derived bioactive compounds in Alzheimer’s disease: An overview of novel insights. *Mar. Drugs* **23**, 1–37. 10.3390/md23070274 (2025). 10.3390/md23070274 PMC12298236 40710500

[36] Jha, B. Z. Oxide Nanoparticles and Their Biomedical Applications. *J. Inorg. Organomet. Polym.* **33**, 1437–1452 (2023). 10.1007/s10904-023-02550-x PMC10118236 37359387

[37] Rehman, H. et al. *Delphinium uncinatum* mediated biosynthesis of zinc oxide nanoparticles and in-vitro evaluation of their antioxidant, cytotoxic, antimicrobial, anti-diabetic, anti-inflammatory, and anti-aging activities. *Saudi J. Biol. Sci.* **30**, 103485. 10.1016/j.sjbs.2022.103485 (2023). 36387032 10.1016/j.sjbs.2022.103485 PMC9649947

[38] Chen, L. L., Guo, J. H., Liu, Y. Y. & Wang, H. Biological responses to combined nanoparticles: Uptake, distribution and toxicity. *Nanomaterials* **16**(11), 695. 10.3390/nano16110695 (2026). 42274702 10.3390/nano16110695 PMC13258702

[39] Bulgarini, A., Lampis, S., Turner, R. J. & Vallini, G. Biomolecular composition of capping layer and stability of biogenic selenium nanoparticles synthesized by five bacterial species. *Microb. Biotechnol.* **14**(1), 198–212. 10.1111/1751-7915.13666 (2021). 33068075 10.1111/1751-7915.13666 PMC7888468

[40] Bhattacharjee et al. Nano-Se as a novel candidate in the management of oxidative stress-related disorders and cancer. *Nucleus* **60**, 137–145 (2017).

[41] Faramarzi, S., Anzabi, Y. & Jafarizadeh-Malmiri, H. Nanobiotechnology approach in intracellular selenium nanoparticle synthesis using Saccharomyces cerevisiae—fabrication and characterization. *Arch. Microbiol.* **202**, 1203–1209. 10.1007/s00203-020-01831-0 (2020). 32077990 10.1007/s00203-020-01831-0

[42] Zhai, C. et al. Construction, characterization, antioxidant activity, and effects on properties in vitro digestion of selenium nanoparticles decorated with *Cyperus esculentus* polysaccharides. *Food Biosci.* **59**, 104062 (2024).

[43] El-Saber, M. Effect of biosynthesized Zn and Se nanoparticles on the productivity and active constituents of garlic subjected to saline stress. *Egypt. J. Desert Res.* **71**, 99–128 (2021).

[44] Ibrahim, S. S. et al. Recent progress in the green synthesis, characterization, and applications of selenium nanoparticles. *BIOI* **5**, 1. 10.15212/bioi-2024-0063 (2024).

[45] Ansari, J. A. et al. Recent advances in the therapeutic applications of selenium nanoparticles. *Mol. Biol. Rep.*10.1007/s11033-024-09598-z (2024). 38796570 10.1007/s11033-024-09598-z PMC11127871

[46] Rudayni, H. A. et al. Biological activities of sargassum algae mediated ZnO and Co-doped ZnO nanoparticles as enhanced antioxidant and anti-diabetic agents. *Molecules*10.3390/molecules28093692 (2023). 37175102 10.3390/molecules28093692 PMC10180528

[47] Kifle, Z. D. & Enyew, E. F. Evaluation of in vivo antidiabetic, in vitro α-amylase inhibitory, and in vitro antioxidant activity of leaves crude extract and solvent fractions of *Bersama abyssinica* Fresen (melianthaceae). *J. Evid.-Based Integr. Med.* **25**, 2515690X20935827. (2020). 32718177 10.1177/2515690X20935827 PMC7388106

[48] Jeong, G. J., Khan, S., Tabassum, N., Khan, F. & Kim, Y. M. Marine-bioinspired nanoparticles as potential drugs for multiple biological roles. *Mar. Drugs*10.3390/md20080527 (2022). 36005529 10.3390/md20080527 PMC9409790

[49] moghaddam, A. Neuro-nutraceutical potential of selenium-enriched spirulina platensis in alleviating streptozotocin-induced cognitive impairment: Modulation of oxidative stress and acetylcholine activity in a rat model of Alzheimer’s disease. *South. Afr. J. Bot.* **142**, 142–153 (2025).

[50] Moodie, L. W. K., Sepčić, K., Turk, T., FrangeŽ, R. & Svenson, J. Natural cholinesterase inhibitors from marine organisms. *Nat. Prod. Rep.* **36**, 1053–1092. 10.1039/c9np00010k (2019). 30924818 10.1039/c9np00010k

[51] Li, Y. et al. The protective effect of selenium nanoparticles in osteoarthritis: In vitro and in vivo studies. *Drug Des. Devel. Ther.* **17**, 1515–1529. 10.2147/DDDT.S407122 (2023). 37249927 10.2147/DDDT.S407122 PMC10216853

[52] Metkin, G. et al. Investigating COX-2 and 5-LOX enzyme-related anti-inflammatory and antioxidant activities and phytochemical features of scutellaria salviifolia benth. *Int. J. Mol. Sci.* **26**, 1–17. 10.3390/ijms26125608 (2025). 10.3390/ijms26125608 PMC12193256 40565073

[53] Tang, P., Huang, R., Zhong, X., Chen, X. & Lei, Y. A comprehensive review on selenium and blood pressure: Recent advances and research perspectives. *J. Trace Elem. Med. Biol.* **88**, 127607. 10.1016/j.jtemb.2025 (2025). 39908739 10.1016/j.jtemb.2025.127607

[54] Kobos, L. et al. Comparison of silver nanoparticle-induced inflammatory responses between healthy and metabolic syndrome mouse models. *J. Toxicol. Environ. Health A*. **83**, 249–268. 10.1080/15287394.2020.1748779 (2020). 32281499 10.1080/15287394.2020.1748779 PMC7493428

[55] Bagci, E. Z., Vodovotz, Y., Billiar, T. R., Ermentrout, G. B. & Bahar, I. Bistability in apoptosis: Roles of Bax, Bcl-2, and mitochondrial permeability transition pores. *Biophys. J.* **90**, 1546–1559. 10.1529/biophysj.105.068122 (2006). 16339882 10.1529/biophysj.105.068122 PMC1367306

[56] Fahimi, S., Oryan, S., Ahmadi, R. & Eidi, A. Downregulation of Bax/Bcl-2 expression during apoptosis in the hippocampus of diabetic male Wistar rats: ameliorative effects of peganum harmala seed extract. *Iran. J. Pharm. Res. IJPR*. **21**, e132071. 10.5812/ijpr-132071 (2022). 36915407 10.5812/ijpr-132071 PMC10007996

[57] Fan, L. et al. Alterations of Bax/Bcl-2 ratio contribute to NaAsO_2_-induced thyrotoxicity in human thyroid follicular epithelial cells and SD rats. *Ecotoxicol. Environ. Saf.* **264**, 115449. 10.1016/j.ecoenv.2023.115449 (2023). 37683429 10.1016/j.ecoenv.2023.115449

[58] Hashem, A. H. & Salem, S. S. Green and eco-friendly biosynthesis of selenium nanoparticles using *Urtica dioica* (stinging nettle) leaf extract: Antimicrobial and anticancer activity. *Biotechnol. J.* **17**, e2100432. 10.1002/biot.202100432 (2022). 34747563 10.1002/biot.202100432

[59] Chen, D. et al. Trends and recent progresses of selenium nanoparticles as novel autophagy regulators for therapeutic development. *Front. Nutr.*10.3389/fnut.2023.1116051 (2023). 36819694 10.3389/fnut.2023.1116051 PMC9931911

[60] Barabadi, H. et al. Efficacy of green nanoparticles against cancerous and normal cell lines: A systematic review and meta-analysis. *IET Nanobiotechnol.* **12**(4), 377–391. 10.1049/iet-nbt.2017.0120 (2018). 29768219 10.1049/iet-nbt.2017.0120 PMC8676322

