# Supplementary material for: In vitro biological potential of green-synthesized nanoparticles from the Egyptian edible bivalve Paratapes undulatus (Born, 1778)
Source: Sci Rep. 2026 Aug 19;16:26045. doi: 10.1038/s41598-026-61397-7 (PMC13490527; doi:10.1038/s41598-026-61397-7)
Supplement: Supplementary file 1 [file 41598_2026_61397_MOESM1_ESM.docx]

**In Vitro Biological Potential of Green-Synthesized Nanoparticles from the Egyptian Edible Bivalve Paratapes undulatus (Born, 1778)**

A

RT:

0.00 - 55.01

0

5

10

15

20

25

30

35

40

45

50

55

Time (min)

0

10

20

30

40

50

60

70

80

90

100

Relative Abundance

15.71

26.22

35.98

51.76

43.08

NL:

1.77E6

TIC MS

B

RT:

0.00 - 55.01

0

5

10

15

20

25

30

35

40

45

50

55

Time (min)

0

10

20

30

40

50

60

70

80

90

100

Relative Abundance

5.57

28.59

22.00

52.29

45.63

37.30

NL:

1.43E9

TIC MS

C

RT:

0.00 - 55.02

0

5

10

15

20

25

30

35

40

45

50

55

Time (min)

0

10

20

30

40

50

60

70

80

90

100

Relative Abundance

5.57

28.72

33.42

46.14

39.48

NL:

1.17E9

TIC MS

D

RT:

0.00 - 55.01

0

5

10

15

20

25

30

35

40

45

50

55

Time (min)

0

10

20

30

40

50

60

70

80

90

100

Relative Abundance

5.57

28.59

22.00

52.29

45.63

37.30

NL:

1.43E9

TIC MS

**Fig. 3.** Total ion chromatograms (TIC) of the GC-MS analysis of (A) *Paratapes undulatus* ethanolic extract, (B) Pu-ZnONPs, (C) Pu-AgNPs, and (D) Pu-SeNPs. Several peaks correspond to bioactive compounds, including metal–porphyrin complexes, silicon- and sulfur-containing molecules, and steroidal derivatives, which contribute to the extract’s observed pharmacological properties.

**Table 1: Major Compounds Identified by GC-MS in Pu Extract**

| **No.** | **RT**  **(min)** | **Compound Name** | **MF** | **MW** | **Area %** |
| --- | --- | --- | --- | --- | --- |
| 1 | 5.54 | Chloroform (Methane, trichloro-) | CHCl₃ | 118 | 3.83 |
| 2 | 8.77 | Pentamethyl- pentaphenyl-cyclopentasiloxane | C35H40O5Si5 | 680 | 5.79 |
| 3 | 9.30 | (2-Nitro-5,10,15,20-tetraphenylporphyrinato) nickel (II) | C44H27N5NiO2 | 715 | 3.88 |
| 4 | 10.05 | Copper tetraphenyl porphyrin | C44H28CuN4 | 675 | 3.88 |
| 5 | 11.77 | (5,10,15,20-tetraphenylporphyrinato) zinc (II) | C44H27DN4Zn | 676 | 4.22 |
| 6 | 13.54 | 1-tert-Butyl-1,4-dihydro-5H-tetrazol-5-one | C5H10N4O | 142 | 3.68 |
| 7 | 16.10 | Bis[(2,4,6-Tri-tert-butylphenyl) amino] phenyl chlorosilane | C42H65ClN2Si | 660 | 4.52 |
| 8 | 17.37 | 2,4-Dibromo-deuteroporphyrin IX dimethyl ester | C32H32Br2N4O4 | 694 | 6.16 |
| 9 | 18.32 | 4,8-Dimethyl-1,5-dithia-2,6-cyclooctadiene | C8H12S2 | 172 | 5.44 |
| 10 | 18.53 | 1,5,9,13-Tetrathia-3,11-cyclohexadecaediol | C12H24O2S4 | 328 | 10.33 |
| 11 | 19.01 | 1-(tert-Butyl)-1,3-dihydro-2h-imidazole-2-thione | C7H12N2O | 140 | 4.18 |
| 12 | 19.12 | (5,10,15,20-tetraphenylporphyrinato) zinc (II | C44H27DN4Zn | 676 | 4.51 |
| 13 | 19.26 | Pentamethyl-pentaphenyl-cyclopentasiloxane | C35H40O5Si5 | 680 | 3.65 |
| 14 | 19.58 | 7-syn-Acetoxy-5-exo-hydroxy-3-exo-methoxy carbonyl  -bicyclo [2.2.1] heptane-2,6-carbolactone | C12H14O7 | 270 | 8.94 |
| 15 | 37.38 | Pregn-5-en-20-one, 3,16-bis[(trimethylsilyl)oxy] | C40H71NO5Si4 | 757 | 6.00 |
| 16 | 38.55 | 1,5-Dichloro-trans-9,10-diethynyl-9,10-dihydroanthracene- | C34H30Cl2O6 | 604 | 3.91 |
| 17 | 40.43 | 2-bis(ethoxycarbonyl)methyl-9(2,3,5-tri-O-(2-methylprop-2-  yl) dimethylsilyloxy-α-D-ribofuranosyl) purine | C35H64N4O8Si3 | 752 | 3.69 |

**Table 2. Major GC–GC-MS-identified compounds in Pu-ZnONP**

| **No.** | **Retention Time (min)** | **Identified Compound** | **MF** | **MW** | **Area (%)** |
| --- | --- | --- | --- | --- | --- |
| 1 | 5.13 | Pentaphenylcyclopentasiloxane | C₃₅H₄₀O₅Si₅ | 680 | 0.23 |
| 2 | 5.22 | Pregn-5-en-20-one (TMS derivative) | C₂₁H₃₂O₂ | 757 | 22.90 |
| 3 | 5.28 | Pentane 3-Ethyl-2,4-dimethyl | C₉H₂₀ | 128 | 26.67 |
| 4 | 5.57 | Cyclobutylmethanol | C5H8O | 84 | 43.19 |
| 5 | 5.93 | Acetonitrile, (dimethylamine) | C4H8N2 |  | 0.19 |
| 6 | 6.24 | 5,10,15,20 tetraphenyl [2 (2) H1] porphyrinato) zinc (II) | C44H28N4Zn | 676 | 0.17 |
| 7 | 6.97 | 3-methyl-3-nitrosobutan-2-one | C5H9NO2 | 115 | 1.03 |
| 8 | 7.08 | 1Bromo2,2,3trimethylcyclopropane | C6H11Br | 162 | 1.72 |
| 9 | 7.17 | nButyl2,2Dicyanopropionate | C9H12N2O2 | 180 | 0.22 |
| 0 | 7.87 | (4S,5S/R)5 Hydroxy. Gamma decalactone | C10H18O3 | 186 | 0.27 |
| 11 | 24.84 | Cyclohexane carboxamide | C12H20N2O3 | 240 | 0.16 |

**Table 3. Major compounds identified in GC–MS analysis of Pu-AgNPs**

| **No.** | **R T (min)** | **Compound Name** | **M F** | **MW** | **Area%** |
| --- | --- | --- | --- | --- | --- |
| 1 | 5.10 | Tetraphenyl porphyrin to dibromo-titanium (IV) | C₄₄H₂₈Br₂N₄Ti | 818 | 5.82 |
| 2 | 5.37 | 7,7-dimethyl-1,4-dioxaspiro [2.4] heptan-5-one | C7H10O3 | 142 | 1.32 |
| 3 | 5.57 | (S)-4-Methyl-γ-butyrolactone | C₅H₈O₂ | 100 | 68.41 |
| 4 | 5.77 | Tetraphenyl porphyrin to dibromo titanium (IV) | C44H28Br2N4Ti | 818 | 0.14 |
| 5 | 5.83 | 1-butene,2-Methyl- | C₅H₁₀ | 70 | 0.12 |
| 6 | 6.05 | 2R,4R)-2,4-dimethylhe xan-1-ol | C8H18O | 130 | 0.72 |
| 7 | 6.30 | Propanenitrile,3, 3'-oxybis- | C6H8N2O | 124 | 0.24 |
| 8 | 6.82 | 1-Butanol | C4H10O | 74 | 0.12 |
| 9 | 7.15 | (2R,3S)-2-[(S)-(Methyl sulfinyl) methyl]-3-propyloxirane | C7H14O2S | 162 | 0.14 |
| 10 | 7.23 | 2-Methyl-1,4-butanedioL | C5H14O2 | 106 | 0.25 |
| 11 | 7.53 | Adipamic acid | C6H11NO3 | 145 | 2.71 |
|  | 8.35 | Ethanone, 1-oxiranyl- | C4H6O2 | 86 | 0.16 |

**Table 4. Major GC-MS-identified compounds in Pu-SeNPs**

| **No.** | **RT (min)** | **Identified Compound** | **Molecular Formula** | **MW** | Area (%) |
| --- | --- | --- | --- | --- | --- |
| 1 | 5.17 | Tetraphenyl porphyrin to dichloro titanium (IV) | C₄₄H₂₈Cl₂N₄Ti | 730 | 5.74 |
| 2 | 5.39 | Butane, 1-chloro | C₆H₉cl | 92 | 70.51 |
|  |  |  |  |  |  |
| 3 | 5.71 | 2-(But-3-en-2-yloxy)-1, 7,7-trimethyl-2-azabicyclo [2.2.1] heptane | C14H11NO | 209 | 0.13 |
| 4 | 6. 35 | Cyclohexanemethanol,2-amino-, cis | C7H15NO | 129 | 0.12 |
| 5 | 6.55 | [3,4,2H2] cyclopent-3-en-1-ol | C₅H6D2O | 84 | 6.94 |
| 6 | 7.03 | 6,6-dimethyl-1-ethynyl-7-oxabicyclo [4.1.0] heptan-2-one | C10H12O2 | 164 | 4.92 |
| 7 | 7.20 | Cyclo-butyl methanol | C5H8O | 84 | 2.48 |
| 8 | 7.53 | 2(3H)-Furanone,  3-acetyldihydro- | C6H8O3 | 128 | 0.10 |
| 9 | 7.71 | 1,1-bis (Tri-deuterio methyl) but-2-yn-1-ol | C6H4D6O | 98 | 0.59 |
| 10 | 7.78 | Butanenitrile | C4H7N | 69 | 0.18 |
| 11 | 8.50 | 2,2-Dichloro-4-methyl-3-pentanone | C6H10CL2O | 168 | 0.13 |
| 12 | 8.76 | 4-(Hexahydroxypyrrolo[1,2-b] isozol-2-yl) furan-2(5H) one | C10H13NO3 | 195 | 0.12 |
| 13 | 8.96 | 2,2-dimethyl-1-nitropropane | C5H11NO2 | 117 | 0.39 |
